# The metaRbolomics Toolbox in Bioconductor and beyond

**DOI:** 10.3390/metabo9100200

**Published:** 2019-09-23

**Authors:** Jan Stanstrup, Corey D. Broeckling, Rick Helmus, Nils Hoffmann, Ewy Mathé, Thomas Naake, Luca Nicolotti, Kristian Peters, Johannes Rainer, Reza M. Salek, Tobias Schulze, Emma L. Schymanski, Michael A. Stravs, Etienne A. Thévenot, Hendrik Treutler, Ralf J. M. Weber, Egon Willighagen, Michael Witting, Steffen Neumann

**Affiliations:** 1 Preventive and Clinical Nutrition, University of Copenhagen, Rolighedsvej 30, 1958 Frederiksberg C, Denmark; 2 Proteomics and Metabolomics Facility, Colorado State University, Fort Collins, CO 80523, USA; cbroeckl@colostate.edu; 3 Institute for Biodiversity and Ecosystem Dynamics, University of Amsterdam, 1098 XH Amsterdam, The Netherlands; r.helmus@uva.nl; 4 Leibniz-Institut für Analytische Wissenschaften—ISAS—e.V., Otto-Hahn-Straße 6b, 44227 Dortmund, Germany; nils.hoffmann@isas.de; 5 Department of Biomedical Informatics, College of Medicine, The Ohio State University, Columbus, OH 43210, USA; ewy.mathe@osumc.edu; 6 Max Planck Institute of Molecular Plant Physiology, 14476 Potsdam-Golm, Germany; naake@mpimp-golm.mpg.de; 7 The Australian Wine Research Institute, Metabolomics Australia, PO Box 197, Adelaide SA 5064, Australia; luca.nicolotti@awri.com.au; 8 Leibniz Institute of Plant Biochemistry (IPB Halle), Bioinformatics and Scientific Data, 06120 Halle, Germany; kpeters@ipb-halle.de (K.P.); hendrik.treutler@ipb-halle.de (H.T.); 9 Institute for Biomedicine, Eurac Research, Affiliated Institute of the University of Lübeck, 39100 Bolzano, Italy; johannes.rainer@eurac.edu; 10 The International Agency for Research on Cancer, 150 cours Albert Thomas, CEDEX 08, 69372 Lyon, France; Salekr@IARC.fr; 11 Department of Effect-Directed Analysis, Helmholtz Centre for Environmental Research—UFZ, Permoserstraße 15, 04318 Leipzig, Germany; tobias.schulze@ufz.de; 12 Luxembourg Centre for Systems Biomedicine, University of Luxembourg, 6 avenue du Swing, L-4367 Belvaux, Luxembourg; emma.schymanski@uni.lu; 13 Eawag, Swiss Federal Institute of Aquatic Science and Technology, Überlandstrasse 133, 8600 Dubendorf, Switzerland; michael.stravs@eawag.ch; 14 CEA, LIST, Laboratory for Data Sciences and Decision, MetaboHUB, Gif-Sur-Yvette F-91191, France; etienne.thevenot@cea.fr; 15 Phenome Centre Birmingham and School of Biosciences, University of Birmingham, Edgbaston, Birmingham B15 2TT, UK; r.j.weber@bham.ac.uk; 16 Department of Bioinformatics—BiGCaT, NUTRIM, Maastricht University, 6229 ER Maastricht, The Netherlands; egon.willighagen@maastrichtuniversity.nl; 17 Research Unit Analytical BioGeoChemistry, Helmholtz Zentrum München, 85764 Neuherberg, Germany; michael.witting@helmholtz-muenchen.de; 18 Chair of Analytical Food Chemistry, Technische Universität München, 85354 Weihenstephan, Germany; 19 German Centre for Integrative Biodiversity Research (iDiv), Halle-Jena-Leipzig Deutscher, Platz 5e, 04103 Leipzig, Germany

**Keywords:** metabolomics, lipidomics, mass Spectrometry, NMR spectroscopy, R, CRAN, bioconductor, signal processing, statistical data analysis, feature selection, compound identification, metabolite networks, data integration

## Abstract

Metabolomics aims to measure and characterise the complex composition of metabolites in a biological system. Metabolomics studies involve sophisticated analytical techniques such as mass spectrometry and nuclear magnetic resonance spectroscopy, and generate large amounts of high-dimensional and complex experimental data. Open source processing and analysis tools are of major interest in light of innovative, open and reproducible science. The scientific community has developed a wide range of open source software, providing freely available advanced processing and analysis approaches. The programming and statistics environment R has emerged as one of the most popular environments to process and analyse Metabolomics datasets. A major benefit of such an environment is the possibility of connecting different tools into more complex workflows. Combining reusable data processing R scripts with the experimental data thus allows for open, reproducible research. This review provides an extensive overview of existing packages in R for different steps in a typical computational metabolomics workflow, including data processing, biostatistics, metabolite annotation and identification, and biochemical network and pathway analysis. Multifunctional workflows, possible user interfaces and integration into workflow management systems are also reviewed. In total, this review summarises more than two hundred metabolomics specific packages primarily available on CRAN, Bioconductor and GitHub.

## 1. Introduction

Metabolomics aims to measure, identify and (semi-)quantify a large number of metabolites in a biological system. The methods of choice are generally Nuclear Magnetic Resonance (NMR) spectroscopy or Mass Spectrometry (MS). The latter can be used directly (e.g., direct infusion MS), but is normally coupled to a separation system such as Gas Chromatography (GC-MS), Liquid Chromatography (LC-MS) or Capillary Electrophoresis (CE-MS). To increase the separation power, multidimensional separation systems are becoming common, such as comprehensive two-dimensional GC or LC (GC×GC, LC×LC) or LC combined with ion mobility spectrometry (LC-IMS) before MS detection. Other detection techniques include Raman spectroscopy, UV/VIS (ultraviolet/visible absorbance spectrophotometric detection—typically with a Diode Array Detector (DAD)) and fluorescence. NMR also benefits from separation techniques, such as LC-MS-NMR or LC-SPE-NMR. Additionally, there are a wide variety of pulse programs commonly used in 1D, and an even bigger set of 2D pulse programs used in metabolomics and for metabolite identification; for a comprehensive review on this, see [1]. A general introduction to metabolomics can be found in textbooks like [2,3,4] or online courses like [5,6].

All of these analytical platforms and methodologies generate large amounts of high-dimensional and complex experimental raw data when used in a metabolomics context. The amount of data, the need for reproducible research, and the complexities of the biological problem under investigation necessitates a high degree of automation and standard workflows in the data analysis. In comparison to vendor software, which is usually not open, open source projects offer the possibility to work in community-driven teams, perform reproducible data analysis and to work with different types of raw data. Many tools and methods have been developed to facilitate the processing and analysis of metabolomics data; most seek to solve a specific challenge in the multi-step data processing and analysis workflow.

This review provides an overview of the metabolomics-related tools that are made available as packages (and a limited number of non-trivial, non-packaged scripts) for the statistics environment and programming language R [7]. We have included packages even if they are not anymore part of current CRAN or Bioconductor, i.e., they are available as archived versions only. We have not included packages described in the literature if they are no longer available for download at all. We did include packages that are currently available, but not yet published in the scientific literature. The package descriptions have been grouped in sections according to the typical steps in the metabolomics data analysis pipeline for different analytical technologies, following the typical workflow steps from MS, NMR and UV data analysis, metabolite annotation, statistical analysis, molecular structure, network and pathway analysis and finally covering packages embracing large parts of the workflow.

### 1.1. Metabolomics Data Processing and Analysis

**Figure 1:**
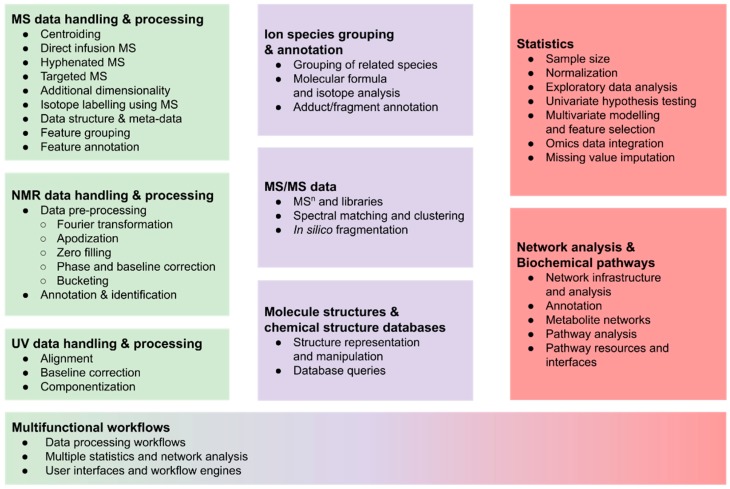
Overview of typical tasks in metabolomics workflows, ranging from metabolite profiling (**left**, green) via metabolite annotation (**center**, purple) to data analysis using statistics and metabolite networks (**right**, red).

The remainder of this section gives a broad overview and explains the typical steps, which are summarised in Figure 1, while common approaches and the available R packages are described in more detail in Section 2.

The first step for any metabolomics study is conversion from vendor formats into open data formats and pre-processing of the obtained raw data. The latter entails converting chromatographic (usually hyphenated to MS) or spectroscopic data into a data matrix suitable for data analysis. For LC-MS data this typically involves feature detection (or peak picking) in individual samples followed by matching of features between samples. For spectroscopic data, this typically means alignment of spectra and potentially binning of the spectra into ‘buckets’. The final matrix will have samples in one dimension and so-called features (unique chromatographic features or spectral bins) in the other dimension. In NMR-based metabolomics, several steps are carried out to process raw time domain data to a spectrum to improve quality such as phasing and baseline correction of the spectrum. Next is alignment of peaks across spectra and samples, followed by segmenting data into bins or a peak fitting step depending on the method used.

Once the analytical data has been pre-processed, it is generally subjected to different statistical approaches to find features that are “interesting” in the context of the experimental design, e.g., differentiating diseased patients from healthy controls. 

In untargeted metabolomics, the selected features contain only the characteristics (e.g., *m*/*z*, retention time, chemical shift, intensity) obtained from the measurement, but not (yet) the metabolite identification or chemical structure as such. Different approaches exist for this metabolite annotation step, ranging from (usually insufficient) database lookup of exact mass (MS) or chemical shift (NMR) alone, to the use of fragmentation patterns obtained in tandem MS experiments, which can be searched against spectral databases or analysed with in silico algorithms, to spectral searching or de novo structure elucidation using combinations of NMR experiments (often 1D and 2D).

Large parts of the metabolomics software landscape in general have been covered in reviews, recent ones include the large list of software packages [8] first described by Spicer et al. [9], and a series of annual reviews covering the list maintained by Misra and others [10,11,12,13], a review by Kannan et al. [14] and the review focussing on approaches for compound identification of LC-MS/MS data by Blaženović et al. [15]. These reviews did include software regardless of the programming environment or language used for the implementation. In Section 2.9 we briefly mention how those can be accessed from within R.

This review will focus on the ecosystem of R packages for metabolomics. It provides an overview of packages to carry out one or multiple of the above-mentioned steps. Some aspects are not covered in depth or not at all. For example, MS-based imaging in metabolomics is an area that has unique challenges and merits its own review, and it is also beyond the scope of this review to discuss all statistical methods that could be applied in metabolomics.

### 1.2. The R Package Landscape

The core of the R language was started in 1997 and provided the basic functionality of a programming language, with some functions targeting statistics. The real power driving the popularity of R today is the huge number of contributed packages providing algorithms and data types for a myriad of application realms. Many packages have an Open Source license. This is not a phenomenon exclusive to R, but is rather a positive cultural aspect of bioinformatics software being mostly published under Open Source license terms, regardless of the implementation language. An R interpreter can be embedded in several other languages to execute R code snippets, and R code can also be executed via different workflow systems (e.g., KNIME or Galaxy, see Section 2.9), which is beneficial for analysis workflows, interoperability and reuse.

These packages are typically hosted on platforms that serve as an umbrella project and are a “home” for the developer and user communities. The Comprehensive R Archive Network (CRAN) repository contains over 14,500 packages for many application areas, including some for bioinformatics and metabolomics. The “CRAN Task Views”, which are manually curated resources describing available packages, books etc., help users navigate CRAN and find packages for a particular task. For metabolomics, the most relevant Task View is “*Chemometrics and Computational Physics*” [16] edited by Katharine Mullen, which includes sections on Spectroscopy, Mass Spectrometry and other tasks relevant for metabolomics applications. The Bioconductor project (BioC for short) was started by a team around Robert Gentleman in 2001 [17], and has become a vibrant community of around 1000 contributors, working on 1741 software, 371 data and 948 annotation packages (BioC release 3.9). In addition to a rich development infrastructure (website, developer infrastructure, version control, build farm, etc.) there are regular workshops for developers and users. To enable reproducible research, BioC runs bi-annual software releases tied to a particular R release, thus ensuring and guaranteeing interoperability of packages within the same BioC release and allowing to install BioC packages from a certain release to reproduce or repeat old data analyses. On both CRAN and BioC, each package has a landing page pointing to sources, build information, binary packages and documentation. On BioC, packages are sorted (by their respective authors) into “BiocViews”, where most packages are targeting genomics and gene expression analysis, and the most relevant ones for metabolomics are Cheminformatics (containing 11 packages), Lipidomics (11), SystemsBiology (66) and, of course, Metabolomics (56). Bioconductor workflows (organised as separate BioC View [18]) provide well documented examples of typical analyses. For community support, BioC maintains mailing lists, a web-based support site, slack communication channels and more. Both CRAN and BioC have a well-defined process for accepting new packages, and the respective developer guidelines (see guidelines for CRAN [19] and for BioC [20]) cover the package life-cycle from submission, updates and maintenance, to deprecation/orphaning of packages. In the case of BioC, new submissions undergo a peer review process, which also provides feedback on technical aspects and integration with the BioC landscape. 

A smaller number of packages are also hosted on sites like rforge.net (https://rforge.net/), r-forge.wu-wien.ac.at (http://r-forge.wu-wien.ac.at/) [21], or sourceforge.net (https://sourceforge.net/) (SF). The non-profit initiative rOpenSci [22] maintains an ecosystem around reproducible research, including staff and community-contributed R packages with additional peer review. Currently, there are no specific metabolomics related packages. 

The GitHub (and also GitLab, Bitbucket) hosting services are not specific to R development, but have gained a lot of popularity due to their excellent support for participation and contribution to software projects. The maintenance of BioC packages on one of the git-based sites has become easier since the BioC team migrated to git as its version control system. A downside of these generic repository hosting sites is that there is no central point of entry, and finding packages for specific tasks is difficult compared with dedicated platforms and relies on search engines and publications. Also, while these hosting services make it easier to provide packages that do not meet BioC and CRAN requirements (e.g., rinchi, due to limitations in the InChI algorithm itself), it also allows users to postpone (or circumvent entirely) the review process that helps ensure the quality of BioC contributions. In addition to generic search engines like Google.com or Bing.com, the rdrr.io is a comprehensive index of R packages and documentation from CRAN, Bioconductor, GitHub and R-Forge. Initially, its main purpose was to find R packages by name, perform full-text search in package documentation, functions and R source code. Recently, it also serves as a hub to actually run R code without local installation, see Section 2.9.

### 1.3. Dependencies and Connectivity of Metabolomics Packages

**Figure 2:**
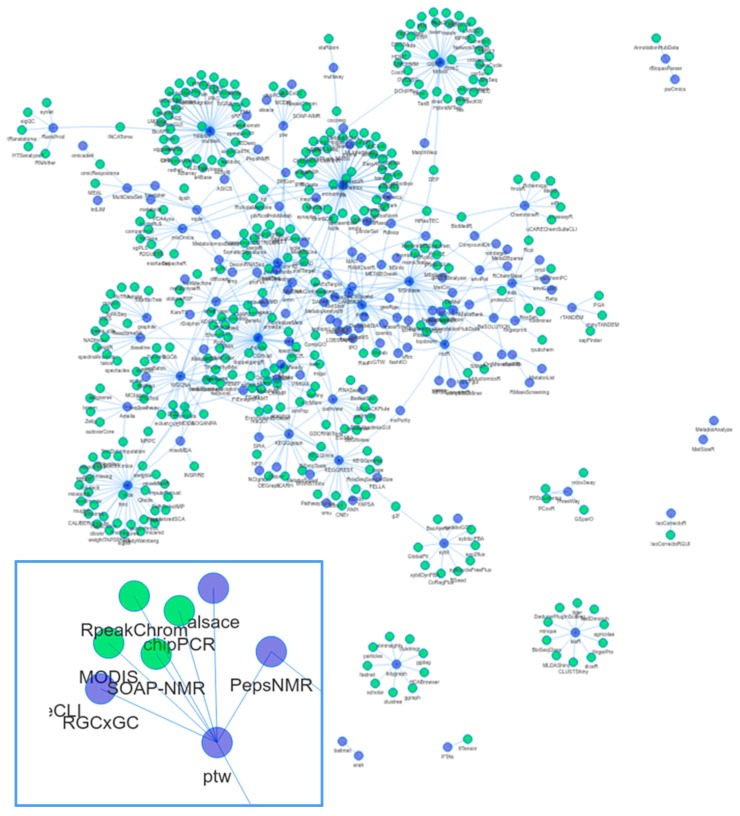
Dependency network of R packages. Shown in blue are packages mentioned in the review. Edges connect to packages that depend on another package, as long as they are in CRAN or BioC. Green nodes correspond to packages in CRAN or BioC not covered in the review. The inset shows the neighbourhood of the ptw package. Not shown are (1) infrastructure packages, e.g., rJava, Rcpp; (2) packages from the review without reverse dependencies; and (3) data packages. Some packages from the review are not in current versions of CRAN or BioC. An interactive version of this figure is also available online (rformassspectrometry.github.io/metaRbolomics-book, Appendix 2) and as Supplemental File S2.

Code reuse and object inheritance can be a sign of a well-connected and interacting community. At the useR!2015 and JSM2015 conferences, A. de Vries and J. Rickert (both Microsoft, London, UK) showed the analysis of the CRAN and BioC dependency network structure [23,24,25]. Compared to CRAN, BioC packages had a higher connectivity: “*It seems that the Bioconductor policy encourages package authors to reuse existing material and write packages that work better together*”. We repeated such an analysis [26] with the packages mentioned in this review and created a network of reverse dependencies (i.e., the set of packages that depend on these metabolomics related packages in BioC or CRAN). The resulting network is shown in Figure 2. 

## 2. R-Packages for Metabolomics

This section reviews packages, relates some of those with similar functionality, and mentions how some of the packages can be used together. The sections in this review are ordered according to specific analytical approaches and the individual required steps.

### 2.1. Mass Spectrometry Data Handling and (Pre-) Processing

For all mass spectrometers, the fundamental data generated is a mass spectrum, i.e., mass-signal intensity pairs. MS-based metabolomics data is typically acquired either as a single mass spectrum or a collection of mass spectra over time, with the time axis (retention time) defined by chromatographic (or other time domain) separation. One of the first steps in metabolomics data processing is usually the reduction of the typically large raw data produced by the instrument to a much smaller set of so-called *features*, which are then subjected to downstream data analysis and interpretation. Features normally represent integrated peaks for a given mass that have been aligned across samples. Establishing these features is called *pre-processing*. The feature detection approaches and packages applicable depend on the type and characteristics of the input data. This section describes the basic data structure for some of the common analytical approaches and shows appropriate tools in R for pre-processing such data, see Table 1 for an overview of the corresponding packages.

#### 2.1.1. Profile Mode and Centroided Data

The mass spectra can be recorded in profile (also called continuum) mode, but are often ‘centroided’. Centroiding is, in effect, a process of peak detection for a profile mode mass spectrum (hence in the *m*/*z* dimension, not in a chromatographic dimension)—a gaussian region of a continuum spectrum with a sufficiently high signal to noise ratio is integrated to give a centroided mass (a “stick” in the mass spectrum as opposed to a continuous signal) and integrated area under the curve. This results in data of reduced size—what was many *m*/*z*-intensity pairs is reduced to a single *m*/*z*-intensity pair. Practically, this reduces the file size considerably, and many data processing tools (e.g., *centWave* in xcms) require MS data that has been centroided. The centroiding can be done either during acquisition on the fly by the instrument software, or as an initial processing step. Post-acquisition centroiding can be performed during conversion of the vendor data format to open formats; typically using msconvert from ProteoWizard [27,28], which in some cases provides access to vendor centroiding algorithms or can alternatively use its own built-in centroiding method. Dedicated vendor tools can also be used, and the R packages MSnbase also provides centroiding capabilities.

#### 2.1.2. Direct Infusion Mass Spectrometry Data

Currently, one of the highest-throughput analytical approaches is direct infusion MS, where the sample is directly injected into the mass spectrometer without any chromatographic separation. This approach can be used with high mass resolution or ultra-high resolution mass spectrometers to discriminate isobaric analytes [29]. Summing or averaging these spectra generates a single mass spectrum, which is representative of that sample. Peak picking can be done using MassSpecWavelet that applies a continuous wavelet transform-based peak detection. xcms provides a wrapper for this function in the *findPeaks.MSW* function. In the Flow Injection Analysis analytical approach (FIA), the sample is transiently injected into the carrier stream flowing directly into the MS instrument. In the absence of chromatographic separation, matrix effects are a challenge for the quantification, especially in complex matrices. FIA coupled to High-Resolution Mass Spectrometry data can be processed with the proFIA workflow which provides efficient and robust peak detection and quantification.

#### 2.1.3. Hyphenated MS and Non-Targeted Data

Chromatographic separation before MS enables better measurement of complex samples and the ability to separate isobaric compounds. Here, the mass spectra are acquired over time as the sample components separate on the chromatography column. The mass spectrum at any given time has the same data structure as any mass spectrum—units of mass to charge ratio and time. As can be inferred from the above descriptions, chromatographically coupled mass spectrometry data is three-dimensional, with dimensions of retention time, *m*/*z*, and intensity. 

For the pre-processing of LC-MS and GC-MS data, xcms is widely used. A recent paper reviewed some of the “xcms family” packages [30], although many more packages exist that build on xcms by providing tools for specialised analyses while others provide improvements of some of the xcms processing steps such as improved peak picking (xMSanalyzer, warpgroup, cosmiq). xcms itself provides several different algorithms for peak picking such as *matchedFilter* [31], *centWave* [32] and *massifquant* [33]. apLCMS, yamss, KPIC2 and enviPick also provide peak picking for LC-MS data independently of xcms. In cases where the alignment of the peak data of different samples is considered (e.g., in cohort studies), xcms and apLCMS include methods to group the peaks by their *m*/*z* and retention times within tolerance levels. The groups are split into sub-groups using density functions and the consensus *m*/*z* and retention time is assigned to each bin.

#### 2.1.4. Targeted Data and Alternative Representations of Data

In addition to the most standard “spectra over time” representation of chromatographically separated MS data, there are several alternative ways to represent the data or simplify the data. The signal intensity for a given mass (or mass range) over chromatographic time can be represented as two equal length vectors, with retention time and intensity as units for the values of those vectors. Examples of these vector pairs include the extracted ion chromatogram (EIC, sometimes also referred to as selected or eXtracted ion chromatogram SIC, XIC), where these chromatograms represent the intensity of a given mass over (retention) time. The data thus contains no spectra, but several SICs. Frequently, this is accomplished by summarising the raw data in a two dimensional matrix consisting of *m*/*z* and time dimensions, with each cell holding the signal intensity for that *m*/*z* and retention time range (or bin). Low mass resolution mass spectrometers often represent the data natively as a SIC and targeted data are also usually represented this way. Recent versions of xcms are also able to process such data, and additional xcms-based functionalities for analysis of targeted data can be found in the packages TargetSearch and SWATHtoMRM, while analysis of isotope labeled data can be found in the packages X^13^CMS, geoRge, and IsotopicLabelling. SIMAT also provides processing for targeted data and does not rely on xcms.

#### 2.1.5. Additional Dimensionality

The vast majority of data collected for metabolomics comprises of three dimensions: retention time, *m*/*z*, and intensity. However, there are more complicated analytical approaches that add additional dimensionality to the data. Two-dimensional chromatography offers two separations in the chromatographic (retention time) domain. The eluent from one column is captured by retention time range and transferred to a second column, where a fast orthogonal separation occurs. When coupled to a mass spectrometer, this generates four-dimensional data (*m*/*z*, first retention time, second retention time, intensity). 

Ion mobility separation (IMS) is a gas phase separation method offering resolution of ions based on molecular shape. This separation occurs on timescales of tens of microseconds, which generates a nested data structure in which there are dozens to hundreds of mass spectra collected across the IMS separation time scale. One can envision this as an ion mobility ‘chromatogram’—however, this chromatogram is nested within the actual chromatographic separation, thus LC-IMS-MS data is also four dimensional. 

Most MS instruments offer the capability to perform selection (or filtering) of ions for fragmentation. The precursor selection can be performed through a quadrupole or ion trap, and fragmentation is often induced by collisions with an inert collision gas. Because this adds a level of mass spectrometry, it is called tandem MS, MS^2^ or MS/MS. Ion trap instruments can further select fragment ions and acquire MS^n^ spectra.

There are several data independent MS/MS approaches, whereby MS/MS precursor selection is done, typically, on a scanning basis. These approaches perform precursor selection in a manner which does not depend on any feedback from the instrument control software or the MS level data. In practice, this precursor window can be either *m*/*z* or ion mobility-based. The processing tools within the R universe (discussed below) are so far underdeveloped for these approaches. With the increased popularity of multidimensional separation, the need for algorithms that can fully utilise the increased separation power is also increasing. 

Currently, osd provides peak picking for unit resolution GC×GC-MS. While the msPeak package provides peak picking for GC×GC-MS data, the peak picking is done on the total ion chromatogram, thus not taking advantage of the mass selectivity provided by the MS detector. It does not appear that any package for R exists that provides peak picking for GC×GC-MS, LC×LC-MS or LC-IMS-MS, similar to (or even better than) commercial tools (e.g., ChromaTOF, GC Image, ChromSquare). Also, at least in the case of GC×GC-MS, unit mass resolution still seems to be the most common use-case, even though high-resolution MS could further improve signal deconvolution and ultimately, analyte identification. Such capabilities are crucial for moving these new powerful analytical approaches into mainstream metabolomics analysis.

#### 2.1.6. Structuring Data and Metadata

The result from the pre-processing is usually a matrix of abundances, rows being features (or features grouped into compounds/molecules) and columns being the samples. Within the statistical community, it is common nowadays to manipulate data matrices with rows as observations and columns as features, this difference stems from the early days, when spreadsheet programs could only handle a limited number of columns smaller than the number of e.g., genes. Such matrices can be easily encapsulated into an *ExpressionSet* class from Bioconductor’s Biobase package [34], the more recent *SummarizedExperiment* defined in the SummarizedExperiment [35] package or the *mSet* class from the metabolomics focussed Metabase [36] package. The main advantage of such objects is their inherent support to align quantitative data along with related metadata (i.e., feature definitions/annotations as row—and sample annotations as column metadata). As an example, a *SummarizedExperiment* can be generated from xcms pre-processing results by adding the output from the *featureValues* function on the xcms result object as quantitative assay and the outputs of the *featureDefinitions* and *pData* functions as row and column annotations, respectively. Many Bioconductor packages for omics data analysis have native support for such objects (e.g., pcaMethods, STATegRa, ropls, biosigner, omicade4).

For the downstream export of mass spectrometry data from metabolomics or lipidomics experiments, the package rmzTab-M provides support for exporting quantitative and identification results backed by analytical and mass spectrometric evidence into the mzTab-M metabolomics file format [37].

**Table 1:** R packages for mass spectrometry data handling and (pre-)processing.

Functionalities	Package	Reference	Repo
**MS Data Handling**			
Parser for common file formats: mzXML, mzData, mzML and netCDF. Usually not used directly by the end user, but provides functions to read raw data for other packages.	mzR	[27]	BioC
Infrastructure to manipulate, process and visualise MS and proteomics data, ranging from raw to quantitative and annotated data.	MSnbase	[38]	BioC
Export and import of processed metabolomics MS results to and from the mzTab-M for metabolomics data format.	rmzTab-M	[37]	GitHub
Converts MRM-MS (.mzML) files to LC-MS style .mzML.	MRMConverteR		GitHub
Infrastructure for import, handling, representation and analysis of chromatographic MS data.	Chromatograms		GitHub
Infrastructure for import, handling, representation and analysis of MS spectra.	Spectra		GitHub
**Peak Picking, Grouping and Alignment (LC-MS Focussed or General)**			
Pre-processing and visualisation for (LC/GC-) MS data. Includes visualisation and simple statistics.	xcms	[31,32]	BioC
Automatic optimisation of xcms parameters based on isotopes.	IPO	[39]	BioC
Parameter tuning algorithm for xcms, MZmine2, and other metabolomics data processing software.	Autotuner		BioC
Pre-processing and visualisation for (LC/GC-) MS data. Includes visualisation and simple statistics.	yamss	[40]	BioC
Peak picking with xcms and apLCMS, low intensity peak detection via replicate analyses. Multi-parameter feature extraction and data merging, sample quality and feature consistency evaluation. Annotation with METLIN and KEGG.	xMSanalyzer	[41]	SF
Pre-processing and alignment of LC-MS data without assuming a parametric peak shape model allowing maximum flexibility. It utilises the knowledge of known metabolites, as well as robust machine learning.	apLCMS	[42]	SF
Peak detection using chromatogram subregion detection, consensus integration bound determination and Accurate missing value integration. Outputs in xcms-compatible format.	warpgroup	[43]	GitHub
Peak picking for (LC/GC-) MS data, improving the detection of low abundance signals via a master map of m/z/RT space before peak detection. Results are xcms-compatible.	cosmiq		BioC
*m/z* detection (i.e., peak picking) for accurate mass data, collecting all data points above an intensity threshold, grouping them by *m/z* values and estimating representative *m/z* values for the clusters; extracting EICs.	AMDORAP	[44]	SF
(GC/LC)-MS data analysis for environmental science, including raw data processing, analysis of molecular isotope ratios, matrix effects, and short-chain chlorinated paraffins.	enviGCMS	[45]	CRAN
Sequential partitioning, clustering and peak detection of centroided LC-MS mass spectrometry data (.mzXML), with Interactive result and raw data plot.	enviPick		CRAN
Peak picking with xcms. Groups chemically related features before alignment across samples. Additional processing after alignment includes feature validation, re-integration and annotation based on custom database.	massFlowR		GitHub
KPIC2 extracts pure ion chromatograms (PIC) via K-means clustering of ions in region of interest, performs grouping and alignment, grouping of isotopic and adduct features, peak filling and Random Forest classification.	KPIC2		GitHub
**Isotope Labeling Using MS**			
Analysis of untargeted LC/MS data from stable isotope-labeling experiments. Also uses xcms for feature detection.	geoRge	[46]	GitHub
Correction of MS and MS/MS data from stable isotope labeling (any tracer isotope) experiments for natural isotope abundance and tracer impurity. Separate GUI available in IsoCorrectoRGUI.	IsoCorrectoR	[47]	BioC
Extension of xcms that provides support for isotopic labeling. Detection of metabolites that have been enriched with isotopic labeling.	X13CMS	[48]	
Analysis of isotopic patterns in isotopically labeled MS data. Estimates the isotopic abundance of the stable isotope (either 2H or 13C) within specified compounds.	IsotopicLabelling	[49]	GitHub
Finding the dual (or multiple) isotope labeled analytes using dual labeling of metabolites for metabolome analysis (DLEMMA) approach, described in Liron [50].	Miso	[51]	CRAN
**Targeted MS**			
Peak picking using peak apex intensities for selected masses. Reference library matching, RT/RI conversion plus metabolite identification using multiple correlated masses. Includes GUI.	TargetSearch	[52]	BioC
Pre-processing for targeted (SIM) GC-MS data. Guided selection of appropriate fragments for the targets of interest by using an optimisation algorithm based on user provided library.	SIMAT	[53]	BioC
Deconvolution of SWATH-MS experiments to MRM transitions.	SWATHtoMRM	[54]	
Automatic analysis of large-scale MRM experiments.	MRMAnalyzer	[55]	
Tailors peak detection for targeted metabolites through iterative user interface. It automatically integrates peak areas for all isotopologues and outputs extracted ion chromatograms (EICs).	AssayR	[56]	GitHub
Targeted peak picking and annotation. Includes shiny GUI.	peakPantheR		GitHub
Toolkit for working with Selective Reaction Monitoring (SRM) MS data and other variants of targeted LC-MS data.	sRm		GitHub
Deconvolution of SWATH-MS data.	DecoMetDIA	[57]	GitHub
Targeted peak picking and annotation. All functions through shiny GUI.	TarMet		GitHub
**GC-MS and GC×GC-MS**			
Unsupervised data mining on GC-MS. Clustering of mass spectra to detect compound spectra. The output can be searched in NIST and ARISTO [58].	MSeasy	[59]	CRAN
Pre-processing for GC/MS, MassBank search, NIST format export.	erah	[60]	CRAN
Pre-processing using AMDIS [61,62] for untargeted GC-MS analysis. Feature grouping across samples, improved quantification, removal of false positives, normalisation via internal standard or biomass; basic statistics.	Metab	[63]	BioC
Deconvolution of GC-MS and GC×GC-MS unit resolution data using orthogonal signal deconvolution (OSD), independent component regression (ICR) and multivariate curve resolution (MCR-ALS).	osd	[64,65]	CRAN
Corrects overloaded signals directly in raw data (from GC-APCI-MS) automatically by using a Gaussian or isotopic-ratio approach.	CorrectOverloadedPeaks	[66]	CRAN
Alignment of GC data. Also GC-FID or any single channel data since it works directly on peak lists.	GCalignR	[67]	CRAN
GC-MS data processing and compound annotation pipeline. Includes the building, validating, and query of in-house databases.	metaMS	[68]	BioC
Peak picking for GC×GC-MS using bayes factor and mixture probability models.	msPeak	[69]	SF
Peak alignment for GC×GC-MS data with homogeneous peaks based on mixture similarity measures.	mSPA	[70]	SF
Peak alignment for GC×GC-MS data with homogeneous and/or heterogenous peaks based on mixture similarity measures.	SWPA	[71]	SF
Chemometrics analysis GC×GC-MS: baseline correction, smoothing, COW peak alignment, multiway PCA is incorporated.	RGCxGC		CRAN
Retention time and mass spectra similarity threshold-free alignments, seamlessly integrates retention time standards for universally reproducible alignments, performs common ion filtering, and provides compatibility with multiple peak quantification methods.	R2DGC	[72]	GitHub
**Flow Injection/Direct Infusion Analysis**			
Pre-processing of data from Flow Injection Analysis (FIA) coupled to High-Resolution Mass Spectrometry (HRMS).	proFIA	[73]	BioC
Flow In-jection Electrospray Mass Spectrometry Processing: data processing, classification modelling and variable selection in metabolite fingerprinting	FIEmspro	[74]	GitHub
Processing Mass Spectrometry spectrum by using wavelet-based algorithm. Can be used for direct infusion experiments.	MassSpecWavelet	[75]	BioC
**Other**			
Filtering of features originating from artifactual interference. Based on the analysis of an extract of E. coli grown in ^13^C-enriched media.	credential	[76]	GitHub
Wrappers for xcms and CAMERA. Also includes matching to a spectral library and a GUI.	metaMS	[68]	BioC
Processing of peaktables from AMDIS, xcms or ChromaTOF. Functions for plotting also provided.	flagme	[77]	BioC
Parametric Time Warping (RT correction) for both DAD and LC-MS.	ptw	[78]	CRAN
R wrapper for X!Tandem software for protein identification.	rTANDEM	[79]	BioC
Building, validation, and statistical analysis of extended assay libraries for SWATH proteomics data.	SwathXtend	[80]	BioC
Split a data set into a set of likely true metabolites and likely measurement artifacts by comparing missing rates of pooled plasma samples and biological samples.	MetProc	[81]	CRAN
Quality of LC-MS and direct infusion MS data. Generates a report that contains a comprehensive set of quality control metrics and charts.	qcrms		GitHub

#### 2.1.7. Ion Species Grouping and Annotation

In MS-based metabolomics, the characterisation and identification of metabolites involves several steps and approaches. After peak (feature) table generation, several tools can be used for grouping features that are postulated to originate from the same molecule. These include the widely used CAMERA for MS^1^ data, as well as RAMClustR (particularly for DIA data), MetTailor, nontarget, CliqueMS and peakANOVA. Packages that support interpretation of the relationship between the ion species, including adducts, isotopes and in-source fragmentation, are InterpretMSSpectrum, CAMERA, nontarget and mzMatch [82]. See Table 2 for a summary of these packages.

Detailed reconstructed isotope patterns can be used to determine the molecular formula of potential candidates. In the case of molecular formula and isotope analysis, the *m*/*z* and intensities for a given (set of) features can be used to calculate a ranked list of possible molecular formulas, based on the accurate mass and relative isotope abundances. The Rdisop, GenFormR and enviPat packages are able to simulate and decompose isotopic patterns into molecular formula candidates. Some post processing can calculate e.g., the double bond equivalents (DBE) and similar characteristics to reduce the number of false positive assignments. Another additional source of information to improve molecular formula estimation is to include MS/MS spectra, as used in MFAssignR, InterpretMSSpectrum or GenFormR.

A typical next step is the annotation of *m*/*z* with putative metabolites using accurate mass lookup, or if the molecular formula was calculated, lookup of the formula in metabolite databases. It has to be noted that annotation with accurate mass search is by no means equivalent to identification. Under the assumption that all the metabolites measured in a sample have some biochemical relation, a global annotation strategy as used in ProbMetab can help as well. Here, the individual ranked lists of formulae are re-evaluated to also maximise the number of pairs with (potential) biochemical substrate-product pairs. The masstrixR package contains several utility functions for accurate mass lookup. This enables matching of measured *m*/*z* values against a given database or library and can additionally perform matching based on retention times (RT) and/or collisional cross sections (CCS) if available.

**Table 2:** R packages for ion species grouping, annotation, molecular formula generation and accurate mass lookup.

Functionalities	Package	Reference	Repos
**Molecular Formula and Isotope Analysis**			
Uses GenForm for molecular formula generation on mass accuracy, isotope and/or MS/MS fragments, as well as performing MS/MS subformula annotation.	GenFormR	[83]	GitHub
Calculation of isotope fine patterns. Also adduct calculations and molecular formula parsing. Web version available at www.envipat.eawag.ch.	enviPat	[84]	CRAN
Molecular formula assignment, mass recalibration, signal-to-noise evaluation, and unambiguous formula selections are provided.	MFAssignR		GitHub
Checking element isotopes, calculating (isotope labelled) exact monoisotopic mass, *m/z* values, mass accuracy, and inspecting possible contaminant mass peaks, examining possible adducts in ESI and MALDI.	MSbox		CRAN
Simulation of and decomposition of Isotopic Patterns.	Rdisop	[85]	BioC
**MS Feature Grouping**			
Grouping of correlated features into pseudo compound spectra using correlation across samples and similarity of peak shape. Annotation of isotopes and adducts. Works as an add-on to xcms.	CAMERA	[86]	BioC
Grouping of features based on similarity between coelution profiles.	CliqueMS	[87]	CRAN
Cluster-based feature grouping for non-targeted GC or LC-MS data.	RAMClustR	[88]	CRAN
Deconvolution of MS/MS spectra obtained with wide isolation windows.	decoMS2	[89]	
Uses dynamic block summarisation to group features belong to the same compound. Correction for peak misalignments and isotopic pattern validation.	MetTailor	[90]	SF
Isotope & adduct peak grouping, homologous series detection.	nontarget	[91]	CRAN
Bayesian approach for grouping peaks originating from the same compound.	peakANOVA	[92]	
Combination of data from positive and negative ionisation mode finding common molecular entities.	MScombine	[93]	CRAN
Grouping of correlated features into pseudo compound spectra using correlation across sample. Annotation of isotopes and adducts. Can work directly with the xcms output.	Astream	[94]	
Navigation of high-resolution MS/MS data in a GUI based on mass spectral similarity.	MetCirc	[95]	BioC
**Ion/Adduct/Fragment Annotation**			
Bayesian probabilistic annotation.	ProbMetab	[96]	GitHub
Isotope & adduct peak grouping, unsupervised homologous series detection.	nontarget	[91]	CRAN
Automatic interpretation of fragments and adducts in MS spectra. Molecular formula prediction based on fragmentation.	InterpretMSSpectrum	[97]	CRAN
Automated annotation using MS/MS data or databases and retention time. Calculation of spectral and chemical networks.	compMS2Miner	[98]	GitHub
Screening, annotation, and putative identification of mass spectral features in lipidomics. Default databases contain ~25,000 compounds.	LOBSTAHS	[99]	BioC
Automated annotation of fragments from MS and MS/MS and putative identification against simulated library fragments of ~500,000 lipid species across ~60 lipid types.	LipidMatch	[100]	GitHub
Annotation of lipid type and acyl groups on independent acquisition-mass spectrometry lipidomics based on fragmentation and intensity rules.	LipidMS	[101]	CRAN
Accurate mass and/or retention time and/or collisional cross section matching.	masstrixR	[102]	GitHub
Downloads KEGG compounds orthology data and wraps the KEGGREST package to extract gene data.	omu	[103]	CRAN
Paired mass distance analysis to find independent peaks in *m/z*-retention time profiles based on retention time hierarchical cluster analysis and frequency analysis of paired mass distances within retention time groups. Structure directed analysis to find potential relationship among those independent peaks. shiny GUI included.	pmd	[104]	CRAN
Pre-processing (xcms), replicate merging, noise, blank and missingness filtering, feature grouping, annotation of known compounds, isotopic labeling analysis, annotation from KEGG or HMDB, common biotransformations and probabilistic putative metabolite annotation using MetAssign.	mzMatch	[82,105]	GitHub
Putative annotation of unknowns in MS^1^ data.	xMSAnnotator	[106]	SF

### 2.2. Metabolite Identification with MS/MS Data

The annotation of features from MS^1^ experiments alone has limited specificity. Additional structural information for metabolite identification is available from tandem MS and higher-order MS^n^ experiments. There are different approaches, ranging from targeted MS/MS experiments and DDA to DIA (e.g., MS^E^, all-ion, broad-band CID, SWATH and other vendor terms). Table 3 provides a summarised overview of R packages for these types of experiments.

#### 2.2.1. MS/MS Data Handling, Spectral Matching and Clustering

Generation of high-quality MS/MS spectral libraries and MS/MS data can be a tedious task. It involves wet lab steps of preparing solutions of reference standards as well as creating MS machine-specific acquisition methods. Several steps can be automated using different R packages presented here.

In case of targeted MS/MS, the instrument isolates specific (specified via method files) masses and fragments them is one possibility. Manually writing targeted MS/MS methods from metabolomics data can be tedious if several tens to hundreds of ions need to be fragmented. The MetShot package supports creating targeted method files for some Bruker and Waters instruments. For all other vendors, optimised lists of non-overlapping peaks (RT-*m*/*z* pairs) can be generated to optimise acquisition in the lowest possible number of methods. 

In data-dependent acquisition (DDA) the instrument is configured to apply a set of rules, which determine which precursor ions are fragmented and MS/MS spectra acquired. DDA approaches also produce a lot of spectra for background peaks or contaminants, which are often of limited use for the purpose of metabolomics studies. Using the RMassBank package, MS^1^ and MS/MS data can be recalibrated and spectra cleaned of artifacts generated. After database lookup of corresponding identifiers, MassBank records are generated.

In data-independent acquisition mode (DIA), the isolation windows are broader, or in some cases, all ions are fragmented, e.g., the Weizmass library [107] is based on MS^E^. The computational challenge for DIA data is to deconvolute the MS/MS data and assign the correct precursor ion. DIA data analysis support is currently being implemented in several R packages. 

MS/MS spectra can be further processed for example by selecting a representative MS/MS spectrum among all spectra associated with a chromatographic peak or by fusing them into a *consensus* spectrum. Subsequently, spectra can be used in downstream analyses such as spectral matching or clustering. Due to the re-use of infrastructure from the MSnbase package, xcms has recently gained native support for MS/MS data handling and hence allows to extract all MS/MS spectra associated with a feature or chromatographic peak for further processing. 

While DDA and DIA are convenient methods, users might miss the accuracy and full control over what is fragmented in the targeted approach. The packages rcdk, MetShot and RMassBank can be combined into a workflow (see [108]) for the generation of records to be uploaded to MS/MS spectral databases (e.g., MassBank [109]) or to be used off-line. MetShot allows the user to specify an arbitrary number RT-*m*/*z* pairs and first sorts them into non-overlapping subsets for which in a second step MS/MS methods (Bruker) or target lists (Agilent, Waters) are generated. It is possible to allow multiple collision energies in a single or separate experiment methods. rcdk was used for calculation of exact masses of adducts. MS/MS data were then acquired on a Bruker maXis plus UHR-Q-ToF-MS. After data collection each run was manually checked for data quality and processed with RMassBank. 

**Table 3:** R packages for MS/MS data.

Functionalities	Package	Reference	Repos
**MS/MS and Libraries**			
Tools for processing raw data to database ready cleaned spectra with metadata.	RMassBank	[110]	BioC
From RT-*m/z* pairs (or *m/z* alone) creates MS/MS experiment files with non-overlapping subsets of the targets. Bruker, Agilent and Waters supported.	MetShot	[111]	GitHub
Creating MS libraries from LC-MS data using xcms/CAMERA packages. A multi-modular annotation function including X-Rank spectral scoring matches experimental data against the generated MS library.	MatchWeiz	[107]	GitHub
Assess precursor contribution to fragment spectrum acquired or anticipated isolation windows using “precursor purity” for both LC-MS(/MS) and DI-MS(/MS) data. Spectral matching against a SQLite database of library spectra.	msPurity	[112]	BioC
Automated quantification of metabolites by targeting mass spectral/retention time libraries into full scan-acquired GC-MS chromatograms.	baitmet	[113]	CRAN
MS/MS spectra similarity and unsupervised statistical methods. Workflow from raw data to visualisations and is interfaceable with xcms.	CluMSID	[114]	BioC
Import of spectra from different file formats such as NIST msp, mgf (mascot generic format), and library (Bruker) to MSnbase objects.	MSnio		GitHub
Multi-purpose mass spectrometry package. Contains many different functions e.g., isotope pattern calculation, spectrum similarity, chromatogram plotting, reading of msp files and peptide related functions.	OrgMassSpecR		CRAN
Annotation of LC-MS data based on a database of fragments.	MetaboList	[115]	CRAN
***In Silico* Fragmentation**			
*In silico* fragmentation of candidate structures.	MetFragR	[116]	GitHub
SOLUTIONS for High ReSOLUTION Mass Spectrometry including several functions to interact with MetFrag, developed during the SOLUTIONS project (www.solutions-project.eu).	ReSOLUTION	[116]	GitHub
Uses MetFrag and adds substructure prediction using the isotopic pattern. Can be trained on a custom dataset.	CCC	[117]	GitHub
**Retention Time Correction**			
Retention time prediction based on compound structure descriptors. Five different machine learning algorithms are available to build models. Plotting available to explore chemical space and model quality assessment.	Retip		GitHub

Spectral matching of measured MS/MS data with spectral libraries is an important step in metabolite identification. Different possibilities for matching of two spectra exist, ranging from simple cosine similarity and the normalised dot product to X-Rank and proprietary algorithms. In MSnbase, different spectra can be compared. Functions for comparison include the number of common peaks, their correlation, their dot product or alternatively a custom comparison function can be supplied. In addition, it will be possible to import spectra from different file formats such as NIST msp, mgf, and Bruker library to MSnbase objects using the MSnio package. MSnbase therefore seems to be the most flexible R package for the computation of spectral similarities. Spectra are binned before comparison. The OrgMassSpecR package contains a simple cosine spectral matching between two spectra. The two spectra are aligned with each other within a defined *m*/*z* error window using one spectrum as the reference. The feature-rich compMS2Miner can import msp files and uses the dot product to calculate the spectral similarity, the msPurity package can perform spectral matching using different similarity functions, and MatchWeiz implements the probabilistic X-Rank algorithm [118].

A growing number of packages, e.g., LOBSTAHS [99], LipidMatch [100] and LipidMS [101], support the annotation of lipids, see Table 2. They use a combination of lipid database lookup, spectral or selected fragment mass matching and in silico spectra prediction. To improve disambiguation between lipids of the same species that may only differ in their fatty acid chain composition, they usually rely on identifying specific MS/MS feature masses that are indicative of substructure fragments, such as the lipid headgroup, the headgroup with a certain fatty acid attached, or losses of fatty acid(s), and other modifications, such as oxidation. Additionally, they require certain intensity ratios between characteristic fragments of a lipid in order to identify the lipid species or subspecies.

#### 2.2.2. Reading of Spectral Databases

NIST msp files and derived msp-like dialects are a commonly used plain text format for the representation of mass spectra. The msp format is described by NIST as part of their Library Conversion Tool [119] documentation, but has many different dialects due to rather loose format definitions. R packages that support the import and export of this file format are able to both use spectral libraries for identification, as well as to create and enrich spectral libraries with new data.

There are various R packages that support the import of NIST msp files (see Table 3), but the support of different dialects varies, e.g., the NIST-like spectral libraries from RIKEN PRIME [120] cannot be parsed by some readers. In addition, none of these packages currently supports the import of additional attributes such as ‘InChIKey: ’ or ‘Collision_energy: ’ as used in the export of MoNA libraries [121]. In essence, most of the packages support the format shown in Listing S1 (see Supplemental File S1, ‘basic NIST’ in Table S1). The metaMS package supports NIST msp files as shown in Listing S2 (see Supplemental File S1, termed ‘canonical NIST’) and RIKEN PRIME provides a similar format with different attributes as shown in Listing S3 (see Supplemental File S1). The packages metaMS, OrgMassSpecR, enviGCMS, and TargetSearch support the export of NIST msp files. The remaining packages partially support the export of results to NIST msp files (see Table S1).

One of the most flexible packages for the handling of NIST msp files is metaMS. This package imports and exports the most attributes, although it does not entirely support generic attributes, and the export is very slow (we observed 20 min for an 8 MB file). In addition, a good library reader should also support mgf (mascot generic format) as available for download from GNPS [122] as well as other common formats such as the MassBank record format and different vendor library formats such as Bruker (.library, another msp flavour) and Agilent (.cef).

### 2.3. NMR Data Handling and (Pre-)Processing 

NMR is another analytical technique commonly used in metabolomics research. The pre-processing steps for NMR data normally include Fourier transformation, apodisation, zero filling, phase and baseline correction, and finally referencing and alignment of spectra. Other steps commonly used are removing the areas without any metabolites such as the water region (from 4.7 to 4.9 ppm), as they generally contain no useful information. There are several R packages that can carry out the above tasks (see Table 4). The PepsNMR and speaq are two examples of such R-based packages. The 1D NMR spectra can then be segmented into spectral regions (also known as bins or buckets) subjected directly to statistical data analysis after a normalisation step. The size of the bins could be fixed or variable (adopted or intelligent binning) based on NMR peaks or even each data point from each peak (full data point resolution) used for data analysis. The NMRProcFlow [123] package provides a graphical and interactive interface for 1D NMR spectral processing and analysis. Additionally, it provides various spectral alignment methods with the ability to use the corresponding experimental-factor levels in a visual and interactive environment, bridging the gap between experimental design and subsequent statistical analyses. Alternatively, peak picking (based on the regions of interest, ROI) can be performed and individual compounds can be identified and integrated prior to statistical analysis. Targeted profiling aims to identify and quantify specific compounds in a sample. The packages that use such approach (ROI) are rDolphin, and rNMR. The bucketed/integrated spectra are normalised to minimise the biological and technical variation. The most common methods are normalisation to a constant sum (e.g., total sum of integral/bin intensities), probabilistic quotient normalisation [124] and dry weight tissue or protein content.

NMR metabolite annotation uses either chemical shifts and multiplicity matching from an existing database, such as Human Metabolome Database [125,126,127,128] (HMDB), a literature experimental search, or uses simulated reference library compounds [129] to match or to fit the existing biological spectra. 1D NMR data often is not sufficient for a confident assignment of the metabolite peaks [130] therefore complementary 2D spectral data acquisition are often required to confirm the assignment [131]. The only package that explicitly deals with 2D NMR is rNMR that takes a targeted approach where the user defines regions of interest to be quantified and compared. DOLPHIN, originally written in MATLAB [132], uses both 1D and 2D NMR data for targeted profiling that is also available as an R version called rDolphin. We are not aware of other R packages that handle 2D NMR data processing. Several general multiway statistical tools such as PARAFAC [133], Tucker3 [134] and MCR have been described [135] that are able to analyse 1D and 2D NMR data, see the section on statistical analysis for a list of packages available for these techniques. BATMAN uses a Bayesian model and some template information such as chemical shifts, *J*-couplings, multiplicity and intensity ratios derived from spectral database to automatically quantify metabolites in a targeted manner [136]. 

**Table 4:** R packages for NMR data handling, (pre-)processing and analysis.

Functionalities	Package	Reference	Repos
**Data Pre-processing and Analysis**			
Interactive environment based on shiny that includes a complete set of tools to process and visualise 1D NMR spectral data using the package Rnmr1D. Processing includes baseline correction, ppm calibration, removal of solvents and contaminants and re-alignment of chemical shifts.	NMRProcFlow	[123]	Bitbucket
Basic processing and statistical analysis steps including several spectral quality assessment as well as pre-processing to multivariate analysis statistics functions.	MetaboMate		GitHub
A tool for processing of 1H NMR data including apodisation, baseline correction, bucketing, Fourier transformation, warping and phase correction. Bruker FID can be directly imported.	PepsNMR	[137]	GitHub
Spectra alignment, peak picking-based processing, quantitative analysis and visualisations for 1D NMR.	speaq	[138,139]	CRAN
**Data Analysis and Identification**			
I dentification and quantification of metabolites in complex 1D 1H NMR spectra.	ASICS	[140]	BioC
Bayesian automated metabolite analyser for 1D NMR spectra. Deconvolution of NMR spectra and automatic metabolite quantification. Also identification based on chemical shift lists.	BATMAN	[136]	RF
Pre-processing and identification in an R-based GUI for 1D NMR.	rDolphin	[141]	GitHub
Analysis of 1D and 2D NMR spectra using a ROI-based approach. Export to MMCD or uploaded to BMRB for identification.	rNMR	[142]	
shiny-based interactive NMR data import and Statistical TOtal Correlation SpectroscopY (STOCSY) analyses.	iSTATS		CRAN
**NMR and Integration with Genomics**			
Handles hyperspectral data, i.e., spectra plus further information such as spatial information, time, concentrations, etc. Such data are frequently encountered in Raman, IR, NIR, UV/VIS, NMR, MS, etc.	hyperSpec		CRAN
MWASTools: an integrated pipeline to perform NMR-based metabolome-wide association studies (MWAS). Quality control analysis; MWAS using various models (partial correlations, generalised linear models); visualisation of statistical outcomes; metabolite assignment using STOCSY; and biological interpretation of MWAS results.	MWASTools	[143]	BioC
An Integrated Suite for Genetic Mapping of Quantitative Variations of 1H NMR-Based Metabolic Profiles. mQTL.NMR provides a complete metabotype quantitative trait locus (mQTL) mapping analysis pipeline for metabolomic data.	mQTL.NMR	[144]	BioC

### 2.4. UV Data Handling and (Pre-)Processing

Another, in metabolomics sometimes under-appreciated, analytical approach is UV absorption detection, usually coupled with an HPLC or UHPLC system. In some cases, the photo-diode array detector (DAD or PDA) is part of an LC-MS system, actually an LC-UV-MS setup. There are other detectors (e.g., fluorescence) with a different principle, but similar characteristics when it comes to the acquired data. Alignment and baseline correction are typically the first steps of pre-processing LC-UV data. Alignment can be achieved for example with the alsace or the ptw package while baseline correction can be achieved using the hyperSpec, ChemoSpec, mdatools (or the baseline packages). The alsace package provides an alternative to using all channels (wavelengths) by first finding unique components (i.e., “pure” spectra) and then performing peak picking in these components. After alignment, general multiway statistical methods like PARAFAC, simultaneous component analysis (SCA), and Tucker Factor Analysis can be applied in the same manner as feature tables would be handled. Table 5 provides an overview of the available R packages for UV data.

**Table 5:** R packages for UV data handling and (pre-)processing.

DAD			
Functionalities	Package	Reference	Repos
Multivariate Curve Resolution (Alternating Least Squares) for DAD data.	alsace	[145]	GitHub
Collection of baseline correction algorithms, along with a GUI for optimising baseline algorithm parameters.	baseline		CRAN
Handles hyperspectral data, i.e., spectra plus further information such as spatial information, time, concentrations, etc. Such data are frequently encountered in Raman, IR, NIR, UV/VIS, NMR, MS, etc.	hyperSpec		CRAN
Projection-based methods for pre-processing, exploring and analysis of multivariate data.	mdatools		CRAN
Parametric Time Warping (RT correction) for both DAD and LC-MS.	ptw	[78]	CRAN

### 2.5. Statistical Analysis of Metabolomics Data

Following the feature detection and grouping steps outlined in the sections above, different paths to statistical analysis are available in R and Bioconductor. Once the “sample versus variable” feature matrix of molecule intensities or abundances has been generated, comprehensive statistical analyses can be performed by using the vast range of packages provided by the R statistical software and the Bioconductor project (see Table 6); see, for instance, StatisticalMethod biocViews [146] and the ExperimentalDesign [147], Cluster [148], Multivariate [149], MachineLearning [150] CRAN Task Views [151]. As mentioned in the introduction, we will only cover common statistical approaches used in metabolomics. Areas such as time-series analysis, clustering methods, machine learning and visualisation of high-dimensional data were dealt with in various books and literature reviews [152,153,154,155,156,157,158,159,160].

With regard to statistical analyses in untargeted metabolomics, two strategies can be differentiated that necessitate the use of different methods. The first strategy “metabolite profiling” is performed by most untargeted metabolomics studies. Here, a bottom-up approach is taken where sets or classes of pre-defined metabolites are studied usually in different phenotypes of the same biological species and differences in metabolites are usually related to more coarse functional or biological levels (e.g., to phenotype or to control vs. treatment in biomedical studies) [161]. Exploratory data analysis, univariate methods, hierarchical clustering (HCA), Principal Component Analysis (PCA) and Multi-Dimensional Scaling (MDS) like methods are very common in metabolite profiling approaches. Feature/variable selection is performed to find only the most significant metabolite candidates that explain the underlying research question, usually using univariate methods to target only specific metabolites that are interesting to the research question of the study [162,163,164,165].

The second strategy, “metabolite fingerprinting”, is commonly used in biomedicine, environmental metabolomics and eco-metabolomics to find metabolite patterns across metabolite profiles. Here, metabolites are characterised without necessarily identifying them, and characterisation usually occurs from spatiotemporally coarser scales to intrinsic scales within biological species [166]. Multivariate statistical methods are used that require reduction of high-dimensional data and, thus, ordination methods are commonly applied like (Orthogonal) Partial Least Squares regression (sometimes also coupled to Discriminant Analysis) ((O)PLS(-DA)), (Linear) Discriminant Analysis ((L)DA), and (Canonical) Correspondence Analysis ((C)CA) that make it possible to relate sets of explanatory variables containing species traits or environmental properties (such as soil type, plant height, smoker/non-smoker, gender, etc.) to the metabolite feature matrix [157,167,168]. Other machine learning methods like Random Forests (RF), Support Vector Machines (SVM) and Neural Networks (NN or ANN) are also applicable [169]. Lately, untargeted metabolomics data is related to other ‘omics using network analysis or Procrustes analysis to visualise (dis)similarities between two or more ‘omics data sets [170,171,172,173].

Extracting a restricted list of features, which still provides a high prediction performance (i.e., a molecular signature), is critical for biomarker validation and clinical diagnostic. Several strategies have been described for feature selection [174,175] (e.g., wrapper approaches such as Recursive Feature Elimination, Genetic Algorithms, or sparse models such as Lasso, Elastic Net, or sparse PLS). Such techniques are implemented in R packages, which also provide detailed comparisons on real datasets in terms of the stability and the size of the selected signature, the prediction performance of the final model, and the computation time [176,177,178,179].

A great number of packages are available for performing statistics on metabolomics datasets. Some of them focus on performing several specific tasks, such as sample size estimation, batch normalisation, exploratory data analysis, univariate hypothesis testing, multivariate modeling and omics data integration. Others, listed in the section ‘Multiple workflow steps’ in Table 6, adopt a more comprehensive approach, providing statistics toolbox that cover different methods and functionalities.

muma is a package designed to be compatible with MS and NMR generated data. The package mainly focuses on performing statistics. It does not contain functions for data extraction and the user has to provide values arranged in a *data.frame* format. The pre-processing is limited to missing value imputation, noise filtering, variable scaling and normalisation. The package also provides tools for outlier detection, univariate and multivariate analysis. Notably, the package offers a script for Statistical TOtal Correlation SpetroscopY (STOCSY) on NMR data.

MOFA proposes tools for the integration of data coming from different omics disciplines (multi-omics). Using factor analysis, it makes it possible to calculate hidden factors that capture the biological sample variation across multi-omics datasets, thus allowing marker discovery. MOFA also provides various tools for the visualisation of results. IntLIM also supports integration of other omics datasets with metabolomics data by leveraging linear modeling to identify gene-metabolite pairs whose relationship differs from one phenotype to another (e.g., positive correlation in one phenotype, negative or no correlation in another). IntLIM includes a user-friendly web interface to perform data quality control of input data, identification of phenotype-dependent gene-metabolite pairs, and interactive visualisation of results. This tool is particularly useful for integrating transcriptomic and metabolomic or other omics data by generating novel hypothesis in a data-driven manner. 

MetaboDiff is presented as an entry-level, user-friendly package for differential metabolomics analysis. The information contained in the input data (metabolomics measurements and metadata) are stored in S4 objects which are used for the downstream processing. The pre-processing consists of missing value imputation, outlier removal and data normalisation, while the data analysis part offers a variety of statistical methods including tools to explore how metabolites relate to each other in sub-pathways.

MetaboAnalystR is a toolbox built over several R packages and contains more than 500 functions organised in eleven modules. The package was created to overcome the limitations of the homonymous web application, such as the possibility of creating flexible customised workflows (including xcms interoperability) and the capacity of dealing with large data sets. MetaboAnalystR functionalities cover a wide range of tools: exploratory statistical analysis, biomarker analysis, power analysis, biomarker meta-analysis, functional enrichment analysis, pathway and joint pathway analysis. Through an implementation of the mummichog algorithm [180], MetaboAnalystR also allows to infer pathways for from user-generated *m*/*z* peak-lists. Using the MetaboAnalyst knowledgebase, MetaboAnalystR provides access to metabolite set libraries, compound libraries and pathway libraries. 

**Table 6:** R packages for statistical analysis of metabolomics data.

Functionalities	Package	Reference	Repos
**Sample Size**			
Estimate sample sizes for metabolomics experiments (NMR and targeted approaches supported).	MetSizeR	[181]	CRAN
**Normalisation**			
Within and between batch correction of LC-MS metabolomics data using either QC samples or all samples.	batchCorr	[182]	GitLab
Drift correction using QC samples or all study samples.	BatchCorrMetabolomics	[183]	GitHub
Cross-contribution robust multiple standard normalisation. Normalisation using internal standards.	crmn	[184]	CRAN
Normalisation using a singular value decomposition.	EigenMS	[185]	SF
Functions for drift removal and data normalisation based on component correction, median fold change, ComBat or common PCA.	intCor	[186]	
Normalisation for low concentration metabolites. Mixed model with simultaneous estimation of a correlation matrix.	Metabnorm	[187]	SF
Multiple fitting models to correct intra- and inter-batch effects.	MetaboQC	[188]	CRAN
Normalisation based on removing unwanted variation [189].	MetNorm	[190]	CRAN
Collection of functions designed to implement, assess, and choose a suitable normalisation method for a given metabolomics study.	NormalizeMets	[191]	CRAN
Support Vector Regression based normalisation and integration for large-scale metabolomics data.	MetNormalizer	[192]	GitHub
A collection of data distribution normalisation methods.	Normalizer	[193]	
Signal and Batch Correction for Mass Spectrometry.	SBCMS		GitHub
**Exploratory Data Analysis**			
Chemometric analysis of NMR, IR or Raman spectroscopy data. It includes functions for spectral visualisation, peak alignment, HCA, PCA and model-based clustering.	ChemoSpec		BioC
Joint analysis of MS and MS/MS data, where hierarchical cluster analysis is applied to MS/MS data to annotate metabolite families and principal component analysis is applied to MS data to discover regulated metabolite families.	MetFamily	[194]	GitHub
A large number of methods available for PCA.	pcaMethods	[195]	BioC
**Univariate Hypothesis Testing ^1^**			
Estimate tail area-based false discovery rates (FDR) as well as local false discovery rates (fdr) for a variety of null models (p-values, z-scores, correlation coefficients, t-scores).	fdrtool	[196]	CRAN
GUI for statistical analysis using linear mixed models to normalise data and ANOVA to test for treatment effects.	MetabR	[197]	RF
Many methods for corrections for multiple testing.	multtest	[198]	BioC
Derives stable estimates of the metabolome-wide significance level within a univariate approach based on a permutation procedure, which effectively controls the maximum overall type I error rate at the α level.	MWSL	[199]	GitHub
**Multivariate Modeling and Feature Selection**			
Find Biomarkers in two class discrimination problems with variable selection methods provided for several classification methods (LASSO, Elastic Net, PC-LDA, PLS-DA, and *t*-test).	BioMark	[178]	CRAN
Recursive feature elimination approach that selects features, which significantly contribute to the performance of PLS-DA, Random Forest or SVM classifiers.	biosigner	[177]	BioC
General framework for building regression and classification models.	caret	[200]	CRAN
Linear and non-linear Discriminant Analysis methods (e.g., LDA), stepwise selection and classification methods useful for feature selection.	klaR	[201]	CRAN
Unsupervised feature extraction specifically designed for analysing noisy and high-dimensional datasets.	KODAMA	[202]	CRAN
Various additions to PCA like PPCA, PPCCA, MPPCA.	MetabolAnalyze	[203]	CRAN
ANOVA-simultaneous component analysis (ASCA), figure of merit, PCA, Goeman’s global test for metabolomic pathways (Q-stat), Penalised Jacobian method (for calculating network connections), time-lagged correlation method and zero slopes method. It also includes centering and scaling functions.	MetStaT		CRAN
Performs variable selection in a multivariate linear model by estimating the covariance matrix of the residuals then use it to remove the dependence that may exist among the responses and eventually performs variable selection by using the Lasso criterion.	MultiVarSel	[204]	CRAN
Fits multi-way component models via alternating least squares algorithms with optional constraints: orthogonal, non-negative, unimodal, monotonic, periodic, smooth, or structure. Fit models include InDScal, PARAFAC, PARAFAC2, SCA, Tucker.	multiway		CRAN
Predictive multivariate modelling using PLS and Random Forest Data. Repeated double cross unbiased validation and variable selection.	MUVR	[179]	GitLab
Probabilistic PLS-DA, Random Forest, SVM, GBM, GLMNET, PAM models for spectral data.	OmicsMarkeR	[176]	BioC
Performs the O2PLS data integration method for two datasets yielding joint and data-specific parts for each dataset.	OmicsPLS	[205]	CRAN
Package for performing Partial Least Squares regression (PLS).	pls	[206]	CRAN
Variable selection methods for PLS, including significance multivariate correlation, selectivity ratio, variable importance in projections (VIP), loading weights, and regression coefficients. It contains also some other modelling methods.	plsVarSel	[207]	CRAN
Decompose a tensor of any order, as a generalisation of SVD also supporting non-identity metrics and penalisations. 2-way SVD is also available. Also includes PCAn (Tucker-n) and PARAFAC/CANDECOMP.	PTAk	[208]	CRAN
Non-parametric method for identifying differentially expressed features based on the estimated percentage of false predictions.	RankProd	[209]	BioC
RF for the construction, optimisation and validation of classification models with the aim of identifying biomarkers. Also includes functionality for normalisation, scaling, PCA, MDS.	RFmarkerDetector		CRAN
Various multivariate methods to analyse metabolomics datasets. Main methods include PCA, Partial Least Squares regression, and extensions like PLS-DA and the orthogonal variants OPLS(-DA).	ropls	[210]	BioC
Fits multi-way component models via alternating least squares algorithms with optional constraints. Fit models include Individual Differences Scaling, Multiway Covariates Regression, PARAFAC (1 and 2), SCA, and Tucker Factor Analysis.	ThreeWay	[211]	CRAN
Contains ordination methods such as ReDundancy Analysis (RDA), (Canonical or Detrended) Correspondence Analysis (CCA, DCA for binary explanatory variables), (Non-metric) MDS and other univariate and multivariate methods. Originally developed for vegetation ecologists, many functions are also applicable to metabolomics.	vegan		CRAN
Biomarker validation for predicting survival. Cross validation methods to validate and select biomarkers when the outcome of interest is survival.	MetabolicSurv		CRAN
Pre-treatment, classification, feature selection and correlation analyses of metabolomics data.	metabolyseR		GitHub
Components search, optimal model components number search, optimal model validity test by permutation tests, observed values evaluation of optimal model parameters and predicted categories, bootstrap values evaluation of optimal model parameters and predicted cross-validated categories.	packMBPLSDA		CRAN
Robust identification of time intervals are significantly different between groups.	OmicsLonDA		BioC
**Omics Data Integration**			
Identifies analyte-analyte (e.g., gene-metabolite) pairs whose relationship differs by phenotype (e.g., positive correlation in one phenotype, negative or no correlation in another). The software is also accessible as a user-friendly interface at intlim.bmi.osumc.edu.	IntLIM	[212]	GitHub
Statistical framework supporting many different types of multivariate analyses, e.g., PCA, CCA, (sparse)PLS(-DA).	mixOmics ggmixOmics	[213] [163]	CRAN
Multi-omics base classes integrable with commonly used R Bioconductor objects for omics data; container that holds omics results.	MultiDataSet	[214]	BioC
Multiple co-inertia analysis of omics datasets (MCIA) is a multivariate approach for visualisation and integration of multi-omics datasets. The MCIA method is not dependent on feature annotation therefore it can extract important features even when they are not present across all datasets.	omicade4	[215]	BioC
STATegRa combines information in multiple omics datasets to evaluate the reproducibility among samples and across experimental conditions using component analysis (omicsNPC implements the NonParametric Combination) and clustering.	STATegRa STATegra-EMS	[216]	BioC
STatistics in R Using Class Templates—Classes for building statistical workflows using methods, models and validation objects.	STRUCT		GitHub
Integration of omics data using multivariate methods such as PLS. Performs community detection and network analysis to allow visualisation of positive or negative associations between different datasets generated using samples from the same individuals. Also available as a shiny app (https://kuppal.shinyapps.io/xmwas).	xMWAS	[217]	GitHub
Joint metabolic model-based analysis of metabolomics measurements and taxonomic composition from microbial communities.	MIMOSA	[218]	GitHub
**Missing Value Imputation**			
Mixture-model for accounting for data missingness’.	metabomxtr	[219]	BioC
Kernel-Based Metabolite Differential Analysis provides a kernel-based score test to cluster metabolites between treatment groups, in order to handle missing values.	KMDA	[220]	CRAN
Visualisation and imputation of missing values. VIM provides methods for the evaluation and visualisation of the type and patterns of missing data. The included imputation approaches are kNN, Hot-Deck, iterative robust model-based imputation, fast matching/imputation based on categorical variables and regression imputation.	VIM	[221]	CRAN
GUI for VIM.	VIMGUI		CRAN
kNN-based imputation for microarray data.	impute	[222]	BioC
Bootstrap-based algorithm and diagnostics for fast and robust multiple imputation for cross sectional, time series or combined cross sectional and time series data.	Amelia	[223]	CRAN
Algorithms and diagnostics for the univariate imputation of time series data.	imputeTS	[224]	CRAN
Methods for the Imputation of incomplete continuous or categorical datasets. missMDA allows missing data imputation using in categorial, continuous or mixed-type datasets using PCA, CA, a multiple correspondence analysis (MCA) model, a multiple factor analysis (MFA) model or factorial analysis for mixed data.	missMDA	[225]	CRAN
Random forest-based missing data imputation for mixed-type, nonparametric data. An out-of-bag (OOB) error estimate is used for model optimisation.	missForest	[226]	CRAN
Multivariate imputation by chained equations using fully conditional specifications for categorical, continuous and binary datasets. It includes various diagnostic plots for the evaluation of the imputation quality.	mice	[227]	CRAN
Missing data imputation using an approximate Bayesian framework. Diagnostic algorithms are included to analyse the models, the assumptions of the imputation algorithm and the multiply imputed datasets.	mi	[228]	CRAN
Iterative Gibbs sampler-based left-censored missing value imputation.	GSimp	[229]	GitHub
**Multiple Workflow Steps**			
Missing value imputation, filtering, normalisation and averaging of technical replications.	MSPrep	[230]	SF
HCA, Fold change analysis, heat maps, linear models (ordinary and empirical Bayes), PCA and volcano plots. Also includes functionality for log transformation, missing value replacement and methods for normalisation. Cross-contribution compensating multiple internal standard normalisation and remove unwanted variation.	metabolomics	[231]	CRAN
Data processing, normalisation, statistical analysis, metabolite set enrichment analysis, metabolic pathway analysis, and biomarker analysis.	MetaboAnalystR	[232,233]	GitHub
Pipeline for metabolomics data pre-processing, with particular focus on data representation using univariate and multivariate statistics. Built on already published functions.	muma	[234]	GitHub
Framework for multi-omics experiments. Identifies sources of variability in the experiment and performs additional analysis (identification of subgroups, data imputation, outlier detection).	MOFA	[235]	BioC
Performs entry-level differential analysis on metabolomics data.	MetaboDiff	[236]	GitHub
STRUCT wrappers (see above) for filtering, normalisation, missing value imputation, glog transform, HCA, PCA, PLS-DA, PLSR, *t*-test, fold-change, ANOVA, Mixed Effects and post-hoc tests.	STRUCTToolbox		GitHub
Data transformation, filtering of feature and/or samples and data normalisation. Quality control processing, statistical analysis and visualisation of MS data.	pmartR		GitHub
Quality control, signal drift and batch correction, transformation, univariate hypothesis testing.	phenomis		GitHub
Missing value filtering and imputation, zero value filtering, data normalisation, data integration, data quality assessment, univariate statistical analysis, multivariate statistical analysis such as PCA and PLS-DA and potential marker selection.	MetCleaning		GitHub
Univariate analysis (linear model), PCA, clustered heatmap, and partial correlation network analysis. Based on classes from the Metabase package [36].	ShinyMetabase		GitHub
Outlier detection, PCA, drift correction, visualisation, missing value imputation, classification.	MetabolomicsBasics	[237]	CRAN
Pre-processing, differential compound identification and grouping, pharmacokinetic parameter calculation, multivariate statistical analysis, correlations, cluster analyses and visualisation.	polyPK	[238]	CRAN

^1^ See also http://www.strimmerlab.org/notes/fdr.html for an exhaustive list of available FDR methods.

### 2.6. Handling of Molecule Structures and Chemical Structure Databases

There are several packages that can deal with cheminformatics tasks, property calculations, metabolite lookup in (web) databases or mapping between databases or structure format conversions (see Table 7). 

A well-established package is rcdk which provide a comprehensive subset of functions from the Chemistry Development Kit [239]. rcdk provides a computer readable representation of molecular structures and provide a wealth of functions to import structures from different molecule structure description formats, manipulate structures, visualise structures and calculate properties and molecular fingerprints. The package fingerprint can then be used to compare fingerprints. rinchi provides reading and writing of InChI and InChIKeys [240]. ChemmineR is an alternative to rcdk, providing many similar functions, with more tools for fingerprints, clustering and others through querying the ChemMine Tools web service [241]. ChemmineR also has significantly faster parsing of SDF files, which can be an advantage when reading large databases. A large number of additional descriptors are available in the package camb which focuses on quantitative predictive models. ChemmineOB provides conversion between a large number of chemical structure formats using OpenBabel [242]. A notable exception is InChI/InChIKey, which is not directly supported by ChemmineOB or ChemmineR and one would thus have to go through rinchi and rcdk for offline import from InChI to ChemmineR or ChemmineOB. RChemMass is a package that combines the functionality of the rcdk with that of RMassBank, and enviPat. The package RRDKit makes (part of) the functionality of the RDKit [243] toolkit available from within R.

Several existing compound databases are useful for metabolomics. These can supply metadata such as common names and synonyms, database identifiers and experimental or predicted properties. The Rpubchem package provides lookup of information available in PubChem [244,245], while the webchem package provide query of a large number of databases including PubChem, ChemSpider [246], Wikidata [247], Chemical Translation Service [248], PHYSPROP [249], Chemical Identifier Resolver [250] and others. BridgeDbR can be used to map identifiers (metabolites, but also genes and proteins, and interactions) between databases, e.g., PubChem to ChemSpider identifiers; RMassBank and RChemMass also provide some useful web-retrieval functions.

The analysis of identified compounds on the level of substance classes can give biochemical insights which are not obvious from the individual structures, or in case the structures are not fully elucidated. The web tool ClassyFire is able to annotate a given structure with compound classes from their ChemOnt taxonomy as well as different substituents [251]. The classyfireR package supports the retrieval of substance classes using the RESTful API of the ClassyFire tool based on InChIKeys.

**Table 7:** R packages for molecule structures and chemical structure databases.

Structure Representation and Manipulation			
Functionalities	Package	Reference	Repos
Subset of functions from the Chemistry Development Kit. Provide a computer readable representation of molecular structures and provide functions to import structures from different molecule structure description formats, manipulate structures, visualise structures and calculate properties and molecular fingerprints.	rcdk	[252]	CRAN
Similar to rcdk in functionality and provides more fingerprints and clustering methods and provides additional tools through querying the ChemMine Tools web service.	ChemmineR	[253]	BioC
Provides conversion of structure representation through OpenBabel.	ChemmineOB		BioC
Exposes functionalities of the RDKit library, including reading and writing of SF files and calculating a few physicochemical properties.	RRDKit		GitHub
Read and write InChI and InChIKey from and to rcdk.	rinchi		GitHub
Maximum Common Substructure Searching using ChemmineR structures.	FmcsR	[254]	BioC
Basic cheminformatics functions tailored for mass spectrometry applications, enhancing functionality available in other packages like rcdk, enviPat, RMassBank etc.	RChemMass		GitHub
Provides fingerprinting methods for rcdk.	fingerprint		CRAN
**Database Queries**			
Calculation of molecular properties.	camb	[255]	GitHub
Querying information from PubChem.	Rpubchem		CRAN
Querying information from various web services (CACTUS, CTS, PubChem, ChemSpider) as part of compound list generation.	RMassBank	[110]	BioC
Querying information from a large number of databases.	webchem	[256]	CRAN
R Interface to the ClassyFire REST API.	classyfireR		CRAN
Allows mapping of identifiers from one database to another, for metabolites, genes, proteins, and interactions.	BridgeDbR		BioC
Define utilities for exploration of human metabolome database, including functions to retrieve specific metabolite entries and data snapshots with pairwise associations.	hmdbQuery		BioC
Parsers for many compound databases including HMDB, MetaCyc, ChEBI, FooDB, Wikidata, WikiPathways, RIKEN respect, MaConDa, T3DB, KEGG, Drugbank, LipidMaps, MetaboLights, Phenol-Explorer, MassBank.	MetaDBparse		GitHub
Functionality to create and use compound databases generated from (mostly publicly) available resources such as HMDB, ChEBI and PubChem.	CompoundDb		GitHub
Standardised and extensible framework to query chemical and biological databases.	biodb		GitHub

### 2.7. Network Analysis and Biochemical Pathways

The R environment offers packages to analyse networks of metabolomics data and metabolic pathways (see Table 8). Within this section, we refer to a ‘pathway’ as a linked series of chemical reactions between molecules, conveyed by enzymes that lead to a product or change in a cell. These molecules are also known as metabolites and transformations occur in the same cellular compartment or in close vicinity. The term ‘network’ refers to the entity of metabolites that are connected biologically, chemically or structurally (e.g., similarity between MS/MS spectra of two metabolites), functionally or by any other measure (e.g., statistically correlated).

#### 2.7.1. Network Infrastructure and Analysis 

The R environment offers a general infrastructure for network analysis. Functionality is implemented in a plethora of software packages, among others igraph, tidygraph or the statnet suite. These packages offer functions to generate networks from respective data input (e.g., adjacency matrices), to analyse networks, calculate network properties and to visualise networks. Generally, any kind of metabolomics data that can be converted to an interpretable format for one of these packages can be analysed by generic network analysis tools. For example, MSnbase offers functionality to calculate similarity scores between MS/MS spectral data that can be readily interpreted as a spectral similarity network (see [257] for the pioneering work of mass spectral molecular networking for biological systems). Such networks can be analysed by the functions provided by the above-mentioned packages or by packages tailored more towards the analysis of biological data (e.g., RedeR). Specifically interesting for metabolomics applications is DiffCorr, an R package to compare correlation networks from two different experimental conditions that builds on an association measure such as Pearson’s correlation coefficient to identify distinctive properties. DiffCorr enables testing of differential correlation of high-dimensional data sets by identifying the first principal component-based ‘eigen-molecules’ in the correlation networks. DiffCorr then tests these differential correlation values based on Fisher’s z-transformation to identify discriminating metabolite pairs that show different response to conditions. Another R package, more tailored towards the analysis of metabolomics data, is BioNetStat, which creates correlation-based networks from metabolite concentration data and analyses the networks based on graph spectra (group of eigenvalues in an adjacency matrix), spectral entropy, degree distribution and node centralities. BioNetStat also allows for KEGG pathway visualisation of metabolite data. 

#### 2.7.2. Metabolite Annotation

As mentioned above in Section 2.2, a major challenge in metabolomics is metabolite annotation, spanning the annotation of known compounds (dereplication) or annotation of unknown metabolites and proposing hypotheses of their structures. Network and pathway analysis can be employed to putatively annotate metabolites in metabolomics data sets. The Bioconductor package MetNet aims at facilitating detection and putative annotation of unknown MS^1^ features in untargeted metabolomic studies. MetNet infers networks by using an ensemble of statistical associations between intensity values across samples and structural information (mass difference matching between features to a list of enzymatic transformation, retention time adjustment) to infer metabolic networks and guide the annotation of especially specialised metabolites of plant, fungi or bacteria samples. Another package for improving annotation is the package xMSAnnotator, which incorporates a multi-criteria scoring algorithm to annotate mass features into different confidence levels. xMSAnnotator uses coelution, pathway level correlations, correlation and KEGG [258,259,260], HMDB, Toxin and Toxin Target Database (T3DB) [261,262], LipidMaps [263] and ChemSpider [246] for annotation and incorporates several filter steps, e.g., by defining modules of co-expressing *m*/*z* features using WGCNA and a topological overlap-based dissimilarity matrix and thereby categorising related metabolites into the same network modules. 

Molecular networking starting from MS/MS data can enhance the annotation of metabolites. MetDNA, implemented in R, JavaScript and Python (available via a web interface on http://metdna.zhulab.cn), combines MS^1^ and MS/MS data to putatively annotate features in metabolomics data sets [264]. MetDNA uses a metabolic reaction network-based recursive algorithm for metabolite annotation employing spectral matching of MS/MS spectra in an automatic fashion. The iterated application of similarity matching between reaction pairs, a substrate metabolite with its product metabolite displaying similar chemical structures, allows the expansion of annotation using seed metabolites or previously annotated metabolites. 

MetCirc, designed for the annotation of MS/MS features in untargeted metabolomics data, visualises the spectral similarity matrix (e.g., the normalised dot product) between MS/MS spectra in a Circos-like interactive shiny application. Within the shiny application, similarity scores can be thresholded, MS/MS spectra can be interactively explored and annotated based on expert knowledge given the similarity score and displayed spectral features. MetCirc relies on the MSnbase framework to store MS/MS spectral data and to calculate similarities between spectra. Similarly, CluMSID employs spectral similarity matching to guide annotation of MS/MS spectra, incorporates functionality to calculate a correlation networks and for hierarchical and density-based clustering. compMS2Miner is another R package for MS/MS feature annotation and offers functionality for noise filtering, MS/MS substructure annotation, calculation of correlation- and spectral similarity-based networks and interactive visualisation. 

#### 2.7.3. Generation of Metabolic Networks 

Several R packages implement the functionality to generate metabolic networks. These networks can subsequently be analysed by their topological properties, be used to identify motifs that differ between experimental conditions or queried to find associations between metabolic features. MetaMapR generates metabolic networks by integrating enzymatic transformation, structural similarity between metabolites, mass spectral similarity and empirical correlation information. Hereby, MetaMapR queries biochemical reactions in KEGG and molecular fingerprints for structural similarities in PubChem. Furthermore, MetaMapR aims at incorporating metabolites with unknown biochemistry and unknown structures, and integrates other data sources (genomic, proteomic, clinical data). The package Metabox offers a pipeline for metabolomics data analysis, including functionality for data-driven network construction using correlation, estimation of chemical structure similarity networks using substructure fingerprints. Its statistical analysis highlights metabolites that are altered based on the experimental design group, which can be further interrogated by network and pathway analysis tools. Furthermore, the package MetabNet includes functionality to perform targeted metabolome-wide association studies (MWAS) and to guide the association of unknowns to a specific metabolic pathway, followed by mapping a target metabolite to the metabolic network structure. 

#### 2.7.4. Pathway Analysis 

Several R packages enable pathway analysis that uses quantitative data of metabolites and maps these to biological pathways. The Bioconductor package pwOmics analyses proteomics, transcriptomics and other-omics data in combination to highlight molecular mechanisms for single-point and time-series experiments. In downstream analyses, pwOmics allows for pathway, transcription factor and target gene identification. 

Another important aspect commonly executed is enrichment analysis to identify pathways that are up- or downregulated given an experimental condition. The R environment offers a whole range of enrichment analysis packages (e.g., tmod for metabolite data). Targeted more towards pathway analysis, FELLA is a Bioconductor package for enrichment analysis. FELLA detects discriminative metabolic features, maps these to known biological pathways of the KEGG database and detects enriched terms by a diffusion algorithm. CePa offers enrichment analysis tools extending conventional gene set enrichment methods by incorporating pathway topologies. CePa takes nodes rather than terms for analysis and uses network centralities as weight of nodes incorporating pathways from the Pathway Interaction Database (PID, [265]), including NCI/Nature Pathway Interaction, BioCarta [266], Reactome [267] and KEGG [258,259,260]. 

MetaboDiff offers functionality to pinpoint to metabolome-wide differences using PCA and t-distributed stochastic neighbor embedding (tSNE) building on the *MultiAssayExperiment* S4 class. Using *t*-test or ANOVA, MetaboDiff identified metabolites that differ in their abundance between groups and identifies modules/sub-pathways by using WGCNA that indicate changes in biological pathways. SDAMS (Semi-parametric differential abundance analysis method for proteomics and metabolomics data from mass spectrometry), building upon the *SummarizedExperiment* S4 class, performs differential abundance analysis on metabolomics data by linking (non-normally distributed) metabolite levels to phenotypic data, containing zero and possibly non-normally distributed non-zero intensity values. 

Many R packages guide the discovery of biomarkers for specific phenotypes. Among these is lilikoi, which maps features to pathways by using standardised HMDB IDs, transforms metabolomic profiles to pathway-based profiles using pathway deregulation scores, a measure how much a sample deviates from a normal level, followed by feature selection, classification and prediction. INDEED (INtegrated DiffErential Expression and Differential network analysis) aims to detect biomarkers by performing a differential expression analysis, which is combined with a differential network analysis based on partial correlation and followed by a network topology analysis. Subsequently, activity scores are calculated based on differences detected in the differential expression and the topology of the differential network that will guide the selection of biomarkers. Another R package for biomarker and feature selection is MoDentify which finds regulated modules, groups of correlating molecules that can span from few metabolites to entire pathways, to a given phenotype. These groups are possibly functionally coordinated, coregulated or driven by a similar or same biological process. Score maximisation using a multivariable linear regression model with the candidate module as dependent and the phenotype and optional covariates as independent variables identifies the modules. Furthermore, MoDentify implements Gaussian graphical models, where depending on the resolution nodes reflect metabolites or entire pathways. 

PAPi (Pathway activity profiling) assigns pathway activity scores to samples to represent the potential pathway activity and statistically detects affected pathways by applying *t*-test or ANOVA. PAPi uses KEGG pathway identifiers. pathwayPCA, with gene selection in mind, offers multi-omics data analysis by estimating sample-specific pathway activities, e.g., taken from the rWikiPathways interface. pathwayPCA takes continuous, binary or survival outcomes as input and estimates contributions of individual genes towards pathway significance. 

R offers packages to analyse metabolic systems and to estimate biochemical reaction rates in metabolic networks using flux balance analysis, e.g., BiGGR, abcdeFBA, sybil, and fbar. For example, BiGGR interfaces with the BiGG databases that contains reconstructions of metabolic networks. After importing pathways from the database, flux balance and downstream routines can be performed, e.g., linear optimisation routines or likelihood-based ensembles of calculated flux distributions fitting experimental data. 

The package MetaboLouise simulates longitudinal metabolomics data. The simulation builds on a mathematical representation that is parameterised according to underlying biological networks, i.e., by defining metabolites and relation between them by initialising enzyme rates. Optionally, the package implements functionality to vary the rates depending on the network state, to add external fluxes and to analyse results based on different parameters. 

#### 2.7.5. Pathway Resources and Interfaces 

A plethora of pathway resources exist, aptly aggregated by Pathguide.org. Several of these resources can be accessed by R packages, which were partly reviewed in [268]: rBiopaxParser, graphite, NCIgraph, pathview, KEGGgraph, SBMLR, rsbml, gaggle, and PSICQUIC. Of these, graphite stores pathway information for proteins and metabolites of currently fourteen species (version 1.28.0). Available databases are KEGG, Biocarta, Reactome, NCI/Nature Pathway Interaction Database, HumanCyc, Panther, SMPDB and PharmGKB. graphite offers in addition topological and statistical pathway analysis tools for metabolomics data by interfaces with the Bioconductor packages SPIA and clipper and supports functionality to build own pathways. Furthermore, RPathVisio enables the creation and editing of biological pathways. RPathVisio makes it possible to visualise data on pathways, to perform statistics on pathway data, and provides an interface to WikiPathways. KEGGREST makes it possible to access the KEGG REST API via a client interface. The package provides utility to search keywords, convert identifiers and link across databases. The package also makes it possible to return amino acid sequences as *AAStringSet* or nucleotide sequences as *DNAStringSet* objects (from the Biostrings [269] package). 

Another package, paxtoolsr, provides literature-curated pathway using the Biological Pathway Exchange (BioPAX) format by providing an interface to the Pathway Commons database (including data from the NCI Pathway Interaction Database (PID), PantherDB, HumanCyc, Reactome, PhosphoSitePlus and HPRD). rWikiPathways is an interface between R and WikiPathways.org. Pathways can be queried, interrogated and downloaded to the R session. Furthermore, rWikiPathways associates metabolite information to pathways when providing the system code of a chemical database (e.g., from HMDB, ChEBI, or ChemSpider).

RaMP provides a relational database of Metabolomics Pathways, integrates pathway, gene, and metabolite annotations from KEGG, HMDB, Reactome, and WikiPathways. The database is downloadable as a standalone MySQL dump, for integration with other software, and is also accessible through an R package, and includes a shiny [270] web interface that supports four basic queries: (1) retrieve analytes (genes of metabolites) given a pathway name; (2) retrieve a pathway for one or more analytes; (3) retrieve analytes involved in the same reaction; (4) retrieve ontologies (cellular location, biofluid locations, etc.) from metabolites. The web interface also supports pathway overrepresentation analysis on genes, metabolites, or genes and metabolites combined (query 3) and includes clustering of significantly enriched pathways according to the percent of overlapping analytes between pathways. Furthermore, the web interface provides network visualisation of gene-metabolites relationships (query 4).

**Table 8:** R packages for network analysis and biochemical pathways.

Functionalities	Package	Reference	Repos
**Network Infrastructure and Analysis**			
Infrastructure for representation of networks, analysis and visualisation.	igraph	[271]	CRAN
Infrastructure for representation of networks, analysis and visualisation.	tidygraph		CRAN
Infrastructure for representation of networks, analysis and visualisation.	statnet		CRAN
Interactive visualisation and manipulation of networks.	RedeR	[272]	BioC
Comparison of correlation networks from two experiments.	DiffCorr	[273]	CRAN
Correlation-based networks from metabolomics data and analysis tools.	BioNetStat		BioC
**Annotation**			
Putative annotation of unknowns in MS^1^ data.	MetNet	[274]	BioC
Putative annotation of unknowns in MS^1^ data.	xMSAnnotator	[106]	SF
Putative annotation of unknowns using MS^1^ and MS/MS data.	MetDNA	[264]	GitHub
Visualisation of spectral similarity networks, putative annotation of unknowns using MS/MS data.	MetCirc	[95]	BioC
Putative annotation of unknowns using MS/MS data, clustering of MS/MS data.	CluMSID	[114]	BioC
Putative annotation of unknowns using MS/MS data.	compMS2Miner	[98]	GitHub
**Generation of Metabolite Networks**			
Biochemical reaction networks, spectral and structural similarity networks.	MetaMapR	[275]	GitHub
Correlation-based networks, structural similarity networks.	Metabox	[276]	GitHub
Targeted metabolome-wide association studies.	MetabNet	[277]	SF
Generation of scale-free correlation-based networks.	WGCNA	[278]	CRAN
**Pathway Analysis**			
Analysis of -omics data, pathway, transcription factor and target gene identification.	pwOmics	[279]	BioC
MSEA a metabolite set enrichment analysis with factor loading in principal component analysis.	mseapca	[280]	CRAN
Enrichment analysis of a list of affected metabolites.	tmod		CRAN
Network-based enrichment analysis of a list of affected metabolites.	FELLA	[281]	BioC
Pathway-based enrichment analysis of a list of affected metabolites.	CePa	[282]	CRAN
Differential analysis, modules/sub-pathway identification using networks.	MetaboDiff	[236]	GitHub
Integrates metabolic networks and RNA-seq data to construct condition-specific series of metabolic sub-networks and applies to gene set enrichment analysis	metaboGSE	[283]	CRAN
Differential analysis.	SDAMS		BioC
Biomarker identification.	lilikoi	[284]	CRAN
Biomarker identification.	INDEED	[285]	BioC
Biomarker identification.	MoDentify	[286]	GitHub
Pathway activity profiling.	PAPi	[287]	BioC
Pathway activity profiling.	pathwayPCA	[288]	BioC
Flux balance analysis.	BiGGR	[289]	BioC
Flux balance analysis.	abcdeFBA		CRAN
Flux balance analysis.	sybil		CRAN
Flux balance analysis.	fbar		CRAN
Identification of affected pathway from phenotype data (interface with graphite).	SPIA	[290]	BioC
Identification of affected pathway from phenotype data (interface with graphite).	clipper		BioC
Interface to PathVisio and WikiPathways and pathway analysis and enrichment.	RPathVisio	[291]	GitHub
Enrichment analysis of a list of genes and metabolites.	RaMP	[292]	GitHub
Simulation of longitudinal metabolomics data based on an underlying biological network	MetaboLouise		CRAN
**Pathway Resources and Interfaces**			
BioPax parser and representation in R.	rBiopaxParser	[293]	BioC
Interface to KEGG, Biocarta, Reactome, NCI/Nature Pathway Interaction Database, HumanCyc, Panther, SMPDB and PharmGKB.	graphite	[294,295]	BioC
Interface to NCI Pathways Database.	NCIgraph		BioC
Interface to KEGG.	pathview	[296]	BioC
Interface to KEGG.	KEGGgraph	[297]	BioC
Interface to systems biology markup language (SBML).	SBMLR		BioC
Interface to systems biology markup language (SBML).	rsbml		BioC
Interface to Gaggle-enabled software (Cytoscape, Firegoose, Gaggle Genome browser).	gaggle		BioC
Interface to molecular interaction databases.	PSICQUIC		BioC
Interface to KEGG REST server.	KEGGREST		BioC
Interface to BioPAX OWL files and the Pathway Commons (PW) molecular interaction database.	paxtoolsr	[298]	BioC
Interface to WikiPathways.	rWikiPathways	[299]	BioC
Database that integrates metabolite and gene biological pathways from HMDB, KEGG, Reactome, and WikiPathways. Includes user-friendly R shiny web application for queries and pathway enrichment analysis.	RaMP-DB	[292]	GitHub

### 2.8. Multifunctional Workflows

When dealing with non-targeted metabolomics data sets, data processing represents a key step for obtaining meaningful and consistent results. While the type and number of data processing methods may vary according to the experimental design and aim of the study, some key steps can be identified that are common for most metabolomics experiments. For this reason, several multifunctional R-based workflows have been developed over the years. A key advantage of using multifunctional workflows is that most of the functions the user needs are available within the same “environment”, so that the data does not have to be formatted to comply with functions in other packages. In this respect, a quite common backbone of R workflows consists in performing a pre-processing step that generates an R object that can be used as argument for different functions. Another advantage is that, in most cases, workflows allow a certain degree of flexibility so that functionalities can be used as standalone functions (modular workflows) to better comply with the user’s needs. The packages covering larger parts of metabolomics workflows available in R are listed in Table 9. 

These multifunctional packages include comprehensive workflows that focus on multiple aspects, such as data pre-processing, data validation, preliminary statistical analysis and data visualisation of large metabolomics datasets. The considered workflows support both MS-based data (LC-MS and GC-MS) and data generated by different analytical platforms. MAIT (Metabolite Automatic Identification Toolkit) offers pre-processing, annotation, statistical analysis and data visualisation. It relies on xcms for peak picking and on CAMERA for the preliminary annotation. In addition to CAMERA, the peak annotation process is implemented by including a functionality that allows relating in-source mass losses to specific biotransformations. Human biotransformations are already included, additional biotransformation criteria can be added by the end user. MAIT also provides several statistical tools and visual representations (e.g., PCA, boxplot, PLS), as well as a function to perform identifications using accurate mass search in HMDB. MetMSLine shows some similarities with MAIT in terms of processing stages (xcms-based pre-processing, multivariate statistics, metabolite identifications). Functionalities characterising MetMSLine include normalisation, signal drift correction using a smoothing method, noise transformation and outlier removal. SimExTargId is a wrapper of different software and R packages for LC-MS data. It includes tools for data conversion (Proteowizard), peak picking and annotation (xcms and CAMERA), outlier detection and data correction (MetMSLine), and basic statistical analysis. A special feature of SimeExTargId is the real time monitoring of the different workflow stages aimed at metabolomics core facilities; users are notified by email in case of processing errors (e.g., outlier detection, signal drift). mzMatch is slightly different from the above-mentioned workflows and is designed to fit in a broader processing pipeline itself. The project also includes a dedicated file format (peakML) and a Java environment. The different modules can still be used independently. mzMatch supports peak picking and grouping using xcms, reproducibility calculation, data normalisation. The peakMonitor app identifies peaks using the local database. The identification is performed on the basis of *m*/*z* and retention time values with user-defined mass accuracy and retention time deviation values. 

MetaDB is built by integrating the metaMS R package into a web application written in Grails. It has also been designed to be integrated with the MetaboLights database. MetaDB supports both LC-MS and GC-MS datasets and offers a wide range of functionalities, including: data storage and metadata management (using the ISA-Tab format and ISACreator tool [300,301]), peak picking and annotation (via metaMS, an xcms and CAMERA add-on) and QC plots. 

MStractor is designed for non-expert users to carry out non-targeted data processing on LC-MS experiments. It gathers xcms and CAMERA functions in a user-friendly pipeline, requiring minimal input and providing graphical QC outputs throughout the workflow. It also includes a manual peak curation step and the possibility of calculating descriptive statistics for each sample class.

patRoon is an interface for different MS-based open source software for non-targeted data processing. patRoon covers different aspects of metabolomics workflows, such as file conversion to open data formats (mzXML and mzML), feature extraction and grouping (using several open software and the R packages xcms, OpenMS, enviPick), extraction of MS and MS/MS data (mzR), component generation (RAMClustR, CAMERA, nontarget), formula calculation (GenForm) and compound identification through automatic annotation of MS/MS spectra (MetFrag and SIRIUS with CSI:FingerID). Other functionalities include (interactive) visualisation and reporting of workflow data, comparison and combining results from different workflow algorithms and several data reduction and selection strategies.

specmine provides a general framework that addresses a variety of different analytical platforms, such as LC-MS, GC-MS, NMR, IR and UV-Vis. The package supports many data formats and includes the possibility of adding metadata in a tabular format. It relies on xcms for LC-MS and GC-MS data pre-processing, on hyperSpec for NMR, IR and UV/VIS data processing and on MAIT for metabolite identification. specmine provides scripts for missing values imputation, univariate and multivariate statistics and machine learning methods. Several case studies are available for testing purposes.

mQTL.NMR is a package specifically for the systematic analysis of 1H NMR metabolomics in quantitative genetics. The package mainly focuses on NMR spectral data pre-processing (normalisation, scaling and peak alignment), mQTL mapping in different model organisms, structural assignment of marker metabolites, and result visualisation.

enviMass is a comprehensive workflow for the data-mining of LC-MS and GC-MS datasets, which also supports MS/MS experiments. It provides the user with a graphical user interface (GUI) and a flexible workflow structure covering common processing steps such as data conversion, peak picking, noise removal,—mass re-calibration, data normalisation, and blank subtraction. It also offers several more specific and advanced functionalities, including isotopologue and adduct grouping, homologous series detection and visualisation, estimation of atom counts for nontarget components, temporal sequences, profile trend detection and processing of both data dependent and data independent acquisition of MS/MS experiments. RMassScreening is a workflow for batch processing of LC-HRMS datasets using a script interface, YAML-based setting configuration and visual interactive data evaluation. It provides wrappers for script-based usage of enviPick and basic enviMass components, and implements suspect screening and combinatorial prediction of possible metabolites (transformation products) from parent compounds. A GUI provides facilities to analyse the results, grouped by sample groups and experimental timepoints, by applying freely adjustable filters. 

MetaboNexus is an interactive data analysis platform for metabolomics experiments, which provides a user friendly R shiny-based GUI designed to work without the need for web server connections. It allows pre-processing (using xcms and MZmine), data scaling, univariate and multivariate statistics (*t*-test, ANOVA, PCA, PLS-DA, Random Forest, heatmap), putative metabolite identification (library matching of MS and MS/MS adduct with METLIN, HMDB and MassBank databases), and several functions for data visualisation. 

**Table 9:** R packages with multifunctional workflows.

Functionalities	Package	Reference	Repos
Convenience wrapper for pre-processing tools (xcms, CAMERA) and several statistical analyses.	MAIT	[302]	BioC
Pre-processing (xcms), replicate merging, noise, blank and missingness filtering, feature grouping, annotation of known compounds, isotopic labeling analysis, annotation from KEGG or HMDB, common biotransformations and probabilistic putative metabolite annotation using MetAssign.	mzMatch	[82,105]	GitHub
xcms and CAMERA-based workflow for non-targeted processing of LC-MS datasets. It includes pre-processing, peak picking, peak filtering, data normalisation and descriptive statistics calculation.	MStractor		GitHub
Performs simultaneous raw data to mzXML conversion (MSConvert), peak picking, automatic PCA outlier detection and statistical analysis, visualisation and possible MS/MS target list determination during an MS1 metabolomic profiling experiment.	simExTargId	[303]	GitHub
Pre-processing of large LC-MS datasets. Performs automatic PCA with iterative automatic outlier removal and, clustering analysis and biomarker discovery.	MetMSLine	[304]	GitHub
Workflow for the systematic analysis of 1H NMR metabolomics dataset in quantitative genetics. Performs pre-processing, mQTL mapping, metabolites structural assignment and offers data visualisation tools.	mQTL.NMR	[144]	BioC
Workflow for pre-processing, quality control, annotation and statistical data analysis of LC-MS and GC-MS-based metabolomics data to be submitted to public repositories.	MetaDB	[305]	GitHub
Framework mainly built on several already published packages. It supports data processing form different analytical platforms (LC-MS, GC-MS, NMR, IR, UV-Vis).	specmine	[306]	GitHub
Common interface for several different MS-based data processing software. It covers various aspects, such as data preparation and data extraction, formula calculation, compound identification and reporting.	patRoon		GitHub
Processing of high resolution of LC-MS data for environmental trend analysis.	enviMass		Zenodo
Workflow for pre-processing of LC-HRMS data, suspect screening, screening for transformation products using combinatorial prediction, and interactive filtering based on ratios between sample groups.	RMassScreening	[307,308]	GitHub
Workflow to perform pre-processing, statistical analysis and metabolite identifications based on database search of detected spectra.	MetaboNexus	[309]	GitHub
shiny-based platform to extract differential features from LC-MS data, includes xcms-based feature detection, statistical analysis, prediction of molecular formulas, annotation of MS/MS spectra, MS/MS molecular networking and chemical compound database search.	METABOseek		GitHub
shiny interface to Metabolomics packages and MetaboAnalyst scripts.	MetaboShiny	[310]	GitHub
Pre-processing and visualising LC-MS data, as well as statistical analyses, mainly based on univariate linear models.	amp		GitHub

### 2.9. User Interfaces and Workflow Management Systems

Visualisation is an important part of data analysis. Traditionally, graphics in R have been focussed on creating static plots, while typical explorative studies generally require interactive visualisation to fully investigate the data. User interactions could range from simply zooming in chromatographic or spectroscopic data through to temporarily excluding data from a complex plot for clarity. Several packages in R are available for making interactive plots, e.g., the Plotly library [311], to create interactive graphics from the static plots generated by the popular plotting framework ggplot2 [312]. The use of interactive plots in R is growing, and is helped by an increasing number of code examples.

Another way interactive plots, and even full GUI tools, are being introduced into R is through the shiny framework, which can create web apps using the full power of R packages as the backend. Many such tools related to metabolomics data analysis are also becoming available, which decreases the learning curve considerably for the typical metabolomics scientist without a computational background. A current gap in the shiny metabolomics landscape are powerful and re-usable widget collections for, e.g., spectra viewers, molecular structures or metabolic networks.

There are several approaches to creating, sharing and using data analysis in R for developers and users, with different strengths and weaknesses. Table 10 summarises several ways to create and run a data analysis with some interpretation and comparative comments. Please note that in some cases it is difficult to quantify “implementation simplicity”, e.g., in the case of shiny apps, which can range from rather straightforward to highly complex. 

**Table 10:** Categorisation of creating and sharing R code and data analysis functionality. Symbols indicate strengths (+, ++) or weaknesses (-, --) or neutral (o) assessment.

Framework	Implementation Simplicity Low to High	User- Friendliness Low to High	Interactivity	Example URLs
R script	++	--	-	write .mzTab
R Markdown vignette	o	o	--	xcms, patRoon
Jupyter Notebook	o	+	+	MSEAp
LearnR (CRAN)	-	++	+	LearnR Examples
shiny app	--	++	++	MetFamily and apps in e.g., RaMP-DB, IntLIM

All of these environments can be run locally or installed on a (local or cloud-based) server. Recently, several initiatives have started to provide publicly available computing resources. Examples are e.g., the previously mentioned rdrr.io, which offers to paste R code into an online console for execution. The console can also be embedded into individual websites. The same project also hosts rnotebook.io, which allows to create and run R notebooks. The shinyapps.io platform operated by RStudio Inc has free and paid options to host shiny apps. The binder project (involving members from large academic institutions and companies (like UC Berkeley, Cal Poly San Luis Obispo, Wild Tree Tech Switzerland, Netflix or Simula Research Lab) is an infrastructure for creating and using shareable, interactive and reproducible data analysis (not only) with R [313] by taking any GitHub repository, turning it into a Docker image and launching it on a cloud service. The package holepunch [314] simplifies preparing an R project for launching on binder. A public instance is the mybinder.org service providing (limited) resources to execute R-based scripts in a hosted Rstudio, Jupyter notebook or applications written with e.g., shiny. The binder infrastructure code is available on GitHub, so that the service can be offered by universities and research groups to its users, lifting the resource limitations of the public instance. 

In some cases, an R package can provide bindings to existing tools and libraries written in other languages (see Table 11). This is, for example, the case for the packages rcdk or MetFragR using the rJava bindings, or mzR, which is a wrapper around the Proteowizard C++ library using the Rcpp package. The fairly new reticulate package provides the corresponding infrastructure to execute Python from R code.

Several workflow systems support workflow nodes and tools that can wrap and execute R code, and in turn build on the huge number of R packages (not only) for metabolomics. In this way, systems like KNIME [315,316] and Galaxy [317,318] also provide a GUI and visual programming using the wrapped R functionality, and possibly combine with tools developed in other programming frameworks.

Galaxy is a web-based environment for omics data analysis [319]. The Workflow4metabolomics.org online Galaxy infrastructure dedicated to metabolomics [318] includes wrappers of xcms, CAMERA, metaMS, proFIA, ropls, biosigner and is open to new contributions. W4M is supported by two national infrastructures: the French Institute of Bioinformatics (www.france-bioinformatique.fr) and the Infrastructure for Metabolomics and Fluxomics (www.metabohub.fr) [320]. Wrapping R code into a Galaxy module is quite straightforward: examples can be found on the toolshed central repository (toolshed.g2.bx.psu.edu) and in the RGalaxy bioconductor package. An additional benefit is that the workflow developers need to ensure seamless data flow through the workflow steps, and often contribute the glue code to bridge the gap between objects and data structures that are not always directly compatible across different packages and softwares, thus also improving interoperability beyond the use in workflow systems. 

Workflows and input/output data can be publicly referenced [321,322] on the Workflow4metabolomics platform, thus enabling fully reproducible research. By using workflow systems, the reuse and reprocessing of data sets is greatly encouraged, as well as the tracking of data provenance [323]. This way, workflows help to boost the FAIR principles that were shaped for data [324].

**Table 11:** Packages to interface R with other languages and workflow environments.

Functionalities	Package	Reference	Repos
Given an R function and its manual page, make the documented function available in Galaxy.	RGalaxy		BioC
Integration of R and C++. Many R data types and objects can be mapped back and forth to C++ equivalents.	Rcpp	[325]	CRAN
Low-Level R to Java Interface.	rJava		CRAN
Interface to ‘Python’ modules, classes, and functions and translation between R and Python objects.	reticulate		CRAN

### 2.10. Metabolomics Data Sets

Sharing of data has become increasingly common, and metabolomics data are available from MetaboLights [326] in the EU, GNPS [122] and Metabolomics Workbench [327] in the US. In the context of this review, we focus instead on data in R packages, which is important for development, unit testing, documentation and user training (see Table 12). While there is no difference in R between software and data packages per se, they are handled differently in the Bioconductor infrastructure and separate views exist. 

There are several data sets with raw data from LC-MS and flow injection analysis, which can be used by the data pre-processing packages in the previous sections. Other packages contain pre-processed data from GC-MS, LC-MS or NMR in the form of peak tables, which are then typically used in statistics packages, network analysis and other downstream analyses. 

**Table 12:** Metabolomics data sets packaged as R packages.

Content	Package	Reference	Repos
**LC-MS**			
12 HPLC-MS NetCDF files (Agilent 1100 LC-MSD SL).	faahKO	[328]	BioC
16 UPLC-MS mzData files (Bruker microTOFq).	mtbls2	[111]	BioC
12 UPLC-MS mzML files (AB Sciex TripleTOF 5600, SWATH mode).	mtbls297	[329]	GitHub
Different raw MS files (LTQ, TripleQ, FTICR, Orbitrap, QTOF) some in different formats (mzML, mzXML, mzData, mzData.gz, NetCDF, mz5). Also mzid format from proteomics.	msdata		BioC
Metadata and DDA MS/MS spectra of 15 narcotics standards (LTQ Orbitrap XL).	RMassBankData	[110]	BioC
183 × 109 peak table.	ropls	[210]	BioC
69 × 5501 peak table.	biosigner	[177]	BioC
40 × 1632 peak table.	BioMark	[178]	CRAN
Raw MS files from a set of blanks and standards that contain common environmental contaminants (acquired with Bruker maXis 4G).	patRoonData		GitHub
Proteomics, metabolomics GC-MS and Lipidomics data from Calu-3 cell culture; 3 mockulum treated and 9 MERS-CoV treated; Time point, 18 h from MassIVE dataset ids MSV000079152, MSV000079153, MSV000079154.	pmartRdata		GitHub
**FIA-MS**			
6 mzML files (human plasma spiked with 40 compounds acquired in positive mode on an orbitrap fusion).	plasFIA		BioC
mzML files (Thermo Exactive) from comparison of leaf tissue from 4 *B. distachyon* ecotypes with Flow-infusion electrospray ionisation-high resolution mass spectrometry (FIE-HRMS). Also includes data sets with 10 technical injections of human urine and another 10 injections from leaf tissue (ecotype ABR1).	metaboData		GitHub
**GC-MS**			
52 × 154 peak table.	pcaMethods	[195]	BioC
**NMR**			
18 × 189 peak table.	MetabolAnalyze		CRAN
33 × 164 peak table.	MetabolAnalyze		CRAN
ASICSdata: 1D NMR spectra for ASICS.	ASICSdata	[140]	BioC

## 3. Conclusions

This review surveyed both the scientific literature and the R landscape for packages relevant to metabolomics research. While it was very easy to find relevant packages in CRAN and even more so in BioC, many packages are scattered across other source code hosting platforms. While GitHub has a concept of topics (see github.com/search?q=topic:metabolomics+topic:r), and crawlers like rdrr.io can find R packages across several platforms, the best findability can be achieved through well-integrated umbrella projects like Bioconductor, which provide additional infrastructure and also improve the community interaction through conferences and workshops. 

This also shows the need for more detailed metadata of the R packages allowing easier mixing and matching of packages, noting that Bioconductor already does a very good job. R packages already have a long-standing history of metadata annotation via their DESCRIPTION and CITATION files. These provide links to other packages (e.g., dependencies and suggestions) and literature describing the package. Exposing package and vignette meta data with semantic approaches will support the community in developing further, more advanced multi-functional workflows for metabolomics. The authors have recently adopted Bioschemas [330] to make metadata more easily findable. For example, efforts to start annotation in vignettes allows the ELIXIR Training eSupport System TeSS (tess.oerc.ox.ac.uk) to pick up newer versions (see this git commit [331]), and efforts are underway to expose content from the DESCRIPTION file as Bioschemas annotations on Bioconductor (see this pull request [332]). These actions greatly contribute to community adoption and encourage the reuse of R-based computational workflows in different use cases [323].

In some cases, software described in the literature was only available “on request”, which in practice often turns out to be not available anymore. This review also did not assess whether the R packages (and their dependencies) could be installed on a current R installation. A recent survey [333] showed how the repeatability of papers using scientific software drops when software is not available or does not install. Issues/bug reports were filed for packages that were found that were not able to be tested on contemporary operating systems. The way out of the (un-)repeatability trap can be expressed in very few, seemingly trivial, rules [334] and hosting the code in the open repositories, if possible with regular builds or even Continuous Integration. As discussed earlier, the metabolomics packages have tighter connections in an established community such as Bioconductor, rather than in other package repositories. In the last few years, Bioconductor packages for metabolomics and proteomics data analysis started converging towards a common mass spectrometry infrastructure, which simplifies interoperability between these packages. Based on experiences from these efforts, the RforMassSpectrometry (RforMassSpectrometry.org) initiative was recently started aiming at providing efficient, thoroughly documented, tested and flexible R software for MS data import, handling and analysis. Significant improvements can thus be expected in the future, simplifying and unifying MS data handling for the benefit of the end users. RforMassSpectrometry also contains the metaRbolomics-book [335], which will be a continuously developed resource with additional examples beyond this review. 

The authors expect that the metaRbolomics landscape will continue its steady growth rate and keeps track of the evolving metabolomics experiments to come.

## Supplementary Materials

The following are available online at https://www.mdpi.com/2218-1989/9/10/200/s1, Supplemental File S1 contains examples for the MSP File Format. Supplemental File S2 contains the HTML version of Figure 1.
