# Supplementary material for: The metaRbolomics Toolbox in Bioconductor and beyond
Source: Metabolites. 2019 Sep 23;9(10):200. doi: 10.3390/metabo9100200 (PMC6835268; doi:10.3390/metabo9100200)
Supplement: Supplementary file 1 [file metabolites-09-00200-s001.zip › Supplemental File S1.pdf]

Supplemental Material:
The metaRbolomics toolbox in Bioconductor and beyond

1. The MSP File Format and package support

```
Name: unknown
Num Peaks:  2
85.345 100; 76.321 50;
```

Listing S1: Minimal example for the basic NIST format.

Name: 1-Methylhistidine<br>Synon: (2S)-2-amino-3-(1-methyl-1H-imidazol-4-yl)propanoic acid<br>SYNON: $:00in-source<br>DB#: HMDB0000001_c_ms_1469<br>InChIKey: BRMWTNUJHUMWMS-LURJTMIESA-N<br>Instrument_type: GC-MS<br>Retention_index: 1807.71<br>Formula: C7H11N3O2<br>MW: 169<br>ExactMass: 169.0851<br>Comments: "column=5%-phenyl-95%-dimethylpolysiloxane capillary column" "derivatization type=2 TMS" "derivatization formula=C13H27N3O2Si2" "derivative mw=313.544" "retention index=1807.71" "retention index type=based on 9 n-alkanes (C10–C36)" "instrument type=GC-MS" "chromatography type=GC" "cas number=332-80-9" "molecular formula=C7H11N3O2" "total exact mass=169.085126592" "InChIKey=BRMWTNUJHUMWMS-LURJTMIESA-N"<br>Num Peaks: 10<br>70 0.014; 71 0.007; 72 0.02; 76 0.008; 77 0.008;<br>78 0.002; 79 0.003; 80 0.005; 81 0.108; 82 0.017;	NAME: Aspartame; LC-ESI-ITFT; MS2; CE<br>PRECURSORMZ: 295.128848<br>PRECURSORTYPE: [M+H]+<br>INSTRUMENTTYPE: LC-ESI-ITFT<br>SMILES: COC(=O)C(CC1=CC=CC=C1)N=C(O)C(N)CC(O)=O<br>INCHIKEY: IAOZJIPTCAWIRG-UHFFFAOYNA-N<br>Ontology: Peptides<br>COLLISIONENERGY: 35<br>FORMULA: C14H18N2O5<br>RETENTIONTIME:<br>IONMODE: Positive<br>Comment: registered in MassBank<br>Num Peaks: 9<br>120.0804 13<br>180.10201 138<br>217.0968 14<br>235.10789 390<br>245.0921 274<br>260.09171 132<br>263.1026 286<br>277.11859 1000<br>278.1022 28
**Listing S2:** Example for the canonical NIST format.	**Listing S3**: RIKEN PRIME msp format example.

Table S1: Overview of MS/MS handling in different R packages. '-' means not available, for the remaining entries see the text above.

package	read msp	write msp	spectral matching and additional information
baitmet			N vs DB; cosine, Stein & Scott composite similarity product
compMS2Miner	NIST, RIKEN PRIME msp	RIKEN PRIME msp	N vs DB; dot product
enviGCMS		basic NIST	
erah	NIST	only result export	N vs DB; cosine
flagme		only result export	
metaMS	NIST	NIST; slow	1 vs DB, N vs DB; proprietary
MatchWeiz			N vs DB; X-Rank
MetCirc			N vs N; normalized dot product; will switch to MSnbase functions soon
MSeasy		only result export	N vs DB; Queries the NIST mass spectral search tool
MSnbase	**	**	1 vs 1, N vs N; dot product and more, user def.
msPurity			N vs DB; dot product
OrgMassSpecR	basic NIST	basic NIST	1 vs 1; normalized dot product
RAMClustR			RAMClustR can import and utilize spectrum similarities from MS-FINDER;
rTANDEM			N vs DB; dot product; R wrapper for X!Tandem software
SwathXtend	- (PeakView / OpenSWATH)	- (PeakView / OpenSWATH)	
TargetSearch	NIST (with error)	NIST	N vs DB; RI-based
